# Risk of liver dysfunction with ACE inhibitors based on real-world data from the MID-NET^®^ in Japan

**DOI:** 10.1038/s41440-025-02390-x

**Published:** 2025-10-10

**Authors:** Yuki Kinoshita, Takashi Ando, Kazuhiro Kajiyama, Hotaka Maruyama, Mei Kohama, Ayumi Tanaka, Yusuke Matsunaga, Chieko Ishiguro, Takahiro Nonaka, Naoya Horiuchi, Taihei Tanaka, Yoshiaki Uyama

**Affiliations:** 1 Office of Pharmacovigilance I, Pharmaceuticals and Medical Devices Agency, Tokyo, Japan; 2 Office of Medical Informatics and Epidemiology, Pharmaceuticals and Medical Devices Agency, Tokyo, Japan; 3 Office of Regulatory Science Research, Center for Regulatory Science, Pharmaceuticals and Medical Devices Agency, Tokyo, Japan; 4 Present Address: Office of Pharmacovigilance Ⅱ, Pharmaceuticals and Medical Devices Agency, Tokyo, Japan; 5 Present Address: Section of Clinical Epidemiology, Department of Data Science, Center for Clinical Sciences, National Center for Global Health and Medicine, Tokyo, Japan; 6 Present Address: Graduate School of Medicine, The University of Tokyo, Tokyo, Japan; 7 Present Address: Center for Regulatory Science, Pharmaceuticals and Medical Devices Agency, Tokyo, Japan

**Keywords:** Angiotensin-converting enzyme inhibitors, Hypertension, Liver dysfunction, Pharmacoepidemiology, Safety measure

## Abstract

Warnings about liver dysfunction in Japanese package inserts vary among angiotensin-converting enzyme (ACE) inhibitors, and risk assessment of liver dysfunction with ACE inhibitors has been limited. To evaluate the risk of liver dysfunction among patients prescribed ACE inhibitors available in Japan, we conducted this study based on the real-world data from MID-NET^®^. We identified patients who were newly prescribed ACE inhibitors between January 1, 2009 and December 31, 2019 and excluded patients with liver dysfunction before the first prescription of ACE inhibitors. To compare the risk of liver dysfunction between the control group (enalapril maleate) and each exposure group, a pairwise Cox proportional hazards model was employed to estimate the hazard ratio (HR) adjusted by inverse probability weighting based on the high-dimensional propensity score. A total of 29,817 patients were identified for analysis in the cohort. Compared with the control group, the HRs (95% confidence interval) were 1.37(0.79–2.38) for captopril, 0.71(0.33–1.54) for alacepril, 0.72(0.55–0.93) for imidapril hydrochloride, 1.08(0.86–1.34) for perindopril erbumine, and 0.69(0.52–0.91) for lisinopril hydrate. The risk of liver dysfunction with ACE inhibitors is unlikely to be a class-effect. Although continuous safety monitoring is necessary for promoting proper use of ACE inhibitors, the results indicate that no additional safety measures are currently required for ACE inhibitors that do not carry a liver dysfunction-related warning in Japan.

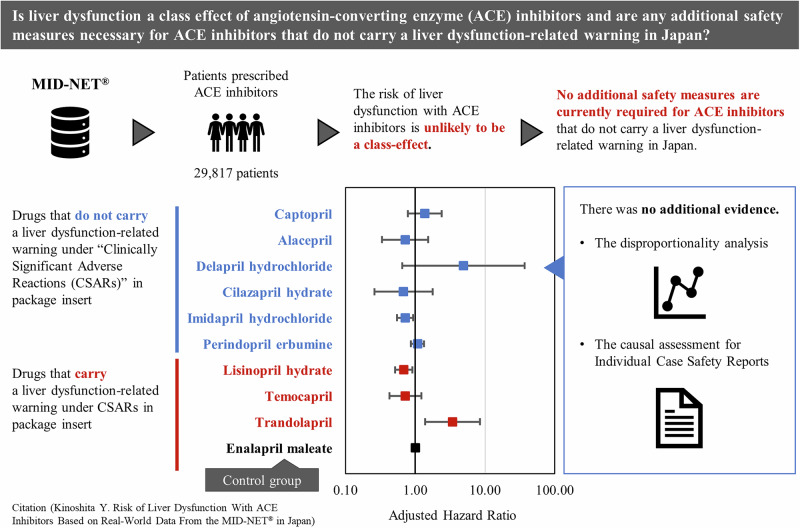

## Introduction

Angiotensin-converting enzyme (ACE) inhibitors are widely prescribed worldwide primarily for the treatment of hypertension and heart failure. In Japan, as with calcium channel blockers, angiotensin II receptor blockers, diuretics, and beta-blockers, ACE inhibitors are considered the first-line treatment for hypertension [1]. However, liver dysfunction is known as one of the adverse reactions to ACE inhibitors. In the United States, the package inserts (PIs) of all ACE inhibitors include warnings related to liver dysfunction under the “WARNINGS AND PRECAUTIONS” section [2]. In contrast, a warning about liver dysfunction under the “Clinically Significant Adverse Reactions” (CSAR) section in Japanese PIs varies among ACE inhibitors; i.e., the warning is included for enalapril maleate, lisinopril hydrate, benazepril hydrochloride, temocapril hydrochloride and trandolapril but not for the others, although liver dysfunction is usually mentioned under the “Other Adverse Reactions” section [3].

Liver dysfunction is attributed to damage of hepatocytes and/or other liver cells by various factors, including drugs. Drug-induced liver injury is a well-known consequence of certain drugs and may lead to severe cases which need medical interventions such as liver transplantation [4]. However, non-drug related factors such as underlying liver disease or other underlying conditions may also be associated with liver dysfunction [5, 6], resulting in difficulty when evaluating this risk based on information only from Individual Case Safety Reports (ICSRs), such as spontaneous adverse event reports. Therefore, in addition to the information in ICSRs, a comparative safety study utilizing the real-world data (RWD) such as electronic medical records will be useful for more precisely evaluating the risk of drug-related liver dysfunction [7].

To compare the risk of liver dysfunction among ACE inhibitors in a real-world setting in Japan and to consider whether any additional safety measures are necessary for ACE inhibitors that do not carry a PI warning, the Pharmaceuticals and Medical Devices Agency (PMDA) conducted a pharmacoepidemiological study to evaluate risk of liver dysfunction with ACE inhibitors available in Japan by utilizing the MID-NET^®^.

Point of view

- Clinical relevance
  The risk of liver dysfunction with ACE inhibitors is unlikely to be a class-effect. No clear increase in the risk of liver dysfunction was observed with ACE inhibitors that do not carry a liver dysfunction-related warning in Japan.
- Future direction
  A study to evaluate the risk of liver dysfunction among patients prescribed ACE inhibitors compared that of patients prescribed other antihypertensive drugs is warranted.
- Considerations for Asian Population
  Although no additional safety measures are currently required for ACE inhibitors without the liver dysfunction-related warnings in Japan, continuous safety monitoring is necessary for promoting proper use of these drugs.

## Methods

### Database

In this study, RWD from MID-NET^®^, a reliable and valuable database in Japan, were used for the analysis because MID-NET^®^ stores electronic medical records, administrative claim data, and diagnosis procedure combination data of more than 5.3 million patients (as of December 2020) in cooperation with 10 healthcare organizations, including 23 university hospitals and regional core hospitals [8, 9]. In this database, data on aspartate aminotransferase (AST), alanine aminotransferase (ALT), alkaline phosphatase (ALP), or total bilirubin (T-Bil), all useful for detecting liver dysfunction, were available for analysis. The study period spanned from 1 January 2009 to 31 December 2019.

Utilization of MID-NET^®^ for this study was approved on October 30, 2020, through a discussion by the expert committee of MID-NET^®^ [10]. The actual data extraction from MID-NET^®^ for analysis was carried out between January 25, 2021, and January 29, 2021. The anonymized dataset was only provided to the authors from the MID-NET^®^ management office. Because this study was conducted as an official activity of the PMDA under the Pharmaceuticals and Medical Devices Agency Law (Article 15–5–(c) and (f)), it was not subject to review by institutional review boards [11, 12].

### Study design and cohort

A new user cohort design was employed to evaluate the risk of liver dysfunction in patients prescribed ACE inhibitors (see Supplementary Fig. S1 for details of the study design). The cohort comprised patients who were newly prescribed at least one ACE inhibitor during the study period (from January 1, 2009 to December 31, 2019). The earliest prescription date within the study period was defined as the cohort entry date (t_0_), and patients with any medical records for 91 days or more before t_0_ were included in the study population. Additionally, patients who met the following criteria during the baseline period (i.e., 90 days before t_0_ to t_0_) were excluded: (1) patients with liver dysfunction defined as grade 2 or higher based on the severity classification of drug adverse events [13] published by the Ministry of Health, Labor and Welfare (MHLW) for AST, ALT, ALP, and T-Bil (i.e., AST ≥ 100 U/L, ALT ≥ 100 U/L, ALP ≥ 805 U/L, or T-Bil ≥ 3.0 mg/dL), (2) patients prescribed anticancer drugs or who underwent radiation therapy, (3) patients prescribed antiviral drugs for hepatitis B or C, and (4) patients whose date of the last medical record was t_0_. Depending on the drug prescribed at t_0_, patients prescribed enalapril maleate were defined as the control group, while patients prescribed one of the other drugs were separately classified into an exposure group. Enalapril maleate was selected as the control because many patients were expected to have prescribed this drug and the warning of liver dysfunction was included under CSAR of this drug’s PI in Japan.

### Follow-up period and outcome definition

The follow-up period for outcome evaluation started 1 day after t_0_ and ended at the earliest date among the following: (1) 30 days after the end of the treatment period, (2) the start date of a different ACE inhibitor prescription, or (3) the date of the last medical record for a patient during the study period. The treatment period comprised the prescription date (start date and duration of prescription) with a 30-day gap period.

The primary outcome was liver dysfunction defined as a case where the following criteria were met based on the severity classification of MHLW [13]: AST, ALT, ALP and T-Bil were all grade 1 or lower (i.e., AST < 100 U/L, ALT < 100 U/L, ALP < 805 U/L, T-Bil < 3.0 mg/dL) during the 90 days before the record date of the condition such as (a) both AST and ALT were grade 2 or higher on the same day (i.e., AST ≥ 100 U/L and ALT ≥ 100 U/L) or (b) both ALP and T-Bil were grade 2 or higher on the same day (i.e., ALP ≥ 805 U/L and T-Bil ≥3.0 mg/dL). The outcome date was defined as the first occurrence of the above criteria during the follow-up period. The secondary outcome was defined as any liver dysfunction of grade 3 or higher on the Common Terminology Criteria for Adverse Events (CTCAE) v5.0 [14, 15].

### Statistical analysis

The patient background, such as sex, age (≥65 years), baseline liver function (normal, grade 1 on the severity classification of MHLW), history of liver dysfunction (normal, grade 1, ≥ grade 2), prescriptions for diabetes medications, prescriptions for dyslipidemia medications, and comorbid heart failure, during the baseline period were tabulated. The median follow-up period with an interquartile range were tabulated for each group. In addition, for the implementation status of liver function tests during the follow-up period, the median numbers of the tests per 100 person-days with an interquartile range were tabulated. Regarding the occurrence of outcomes, the number and percentage of patients with the outcome during the follow-up period were calculated for each group. For the duration to the occurrence of outcome, the median duration with an interquartile range were also calculated for each group.

For comparison of each exposure group with the control group, covariates were selected using the high-dimensional propensity score (HDPS) method for each pair of exposure and control groups [16–18], incorporating the aforementioned patient background as basic covariates. We defined 3 dimensions assessing disease, procedure, and medication information in the baseline period. The coding systems used in each dimension were the first 5 digit codes of the anatomical therapeutic chemical (ATC) [19] for medications, the first 3 digit codes of the International Statistical Classification of Diseases and Related Health Problems 10th revision (ICD-10) [20] for diseases, and the first 4 digit codes of the Japanese Procedure (including administrative fee, surgery and procedure, etc) [21] for medical procedures, with no restrictions on the number of candidate covariates extracted from each dimension. To account for the positivity assumption, covariates with zero patients in either the exposure or control group were excluded. Finally, covariates amounting to one-tenth of the patient number in the exposure group were selected using Bross’s formula with zero-cell correction. Multivariable logistic regression was used to calculate the HDPS, and inverse probability weighting (IPW) was applied for adjustment. To compare the risk of liver dysfunction between the control group (enalapril maleate) and each exposure group, a pairwise Cox proportional hazards model was employed to estimate the crude and adjusted hazard ratio (aHR) along with 95% confidence interval (CI). Truncation was performed at the 1st and 99th percentiles of the weights based on the HDPSs.

Sensitivity analyses were also conducted, including (1) analyses changing the number of selected covariates (250 and 500 covariates), (2) analyses restricted to populations with an overlapping HDPS between the exposure and control group for each pair, to confirm the validity of the primary analysis.

SAS version 9.4 (SAS Institute, Cary, NC, USA) was used for all analyses.

### Additional assessment based on ICSRs

For regulatory decision making, all available scientific evidence and information, including results from pharmacoepidemiological studies, ICSRs, and literatures as well as safety measures taken by foreign regulatory agencies, are used for assessing drug safety and considering necessary safety measures [22]. From that perspective, we also conducted a disproportionality analysis based on data from ICSRs of the World Health Organization (WHO) global database (VigiBase) [23] as of May 23, 2024 for ACE inhibitors that do not carry a liver dysfunction-related CSAR warning in Japan (i.e., captopril, alacepril, delapril hydrochloride, imidapril hydrochloride, and perindopril erbumine) to more comprehensively understand the risk. It should be noted that the information in the VigiBase does not represent the opinion of the Uppsala Monitoring Center or the WHO and comes from a variety of sources, and the probability that the suspected adverse reaction is drug-related is not the same in all cases. Data for ICSRs of drug-related liver dysfunction were extracted by using VigiLyze software targeted for a standardized Medical Dictionary for Regulatory Activities v27.0 query (SMQ) “Drug-related hepatic disorders-comprehensive search”. Information Component (IC) was calculated and signal was recognized as a positive when the lower limit of the 95% CI of IC (IC_0.25_) exceeded 0 [24, 25].

## Results

**Fig. 1:**
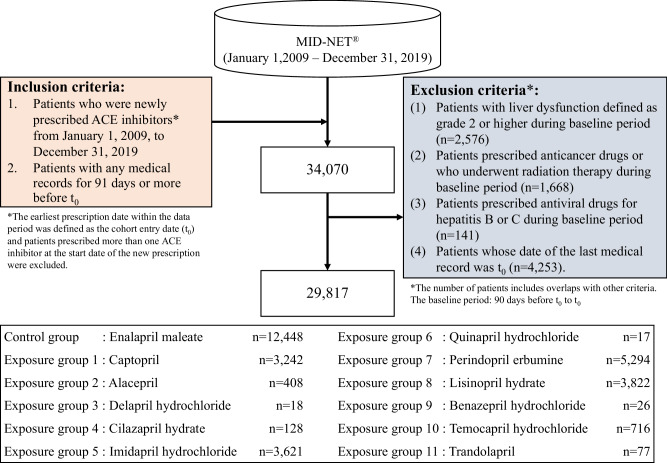
Flow chart for patient selection

A total of 34,070 patients were identified in the cohort including patients newly prescribed an ACE inhibitor during the study period. Of these, 29,817 were included in the analysis after applying all the inclusion and exclusion criteria (Fig. 1. Flow chart for patient selection). The group with the highest number of patients was the control group (enalapril maleate) (*n* = 12,448), followed by the group for perindopril erbumine (*n* = 5294). Many groups had a higher proportion of males and patients aged 65 and older. Liver function at the baseline period (the day closest to t_0_) were normal in many patients in each group (79% or over) and the proportion of patients without a history of liver dysfunction in each group was approximately 70–90% (see Supplementary Table S1 for details of patient background in each group).

In the analysis, the absolute standardized mean differences (ASMD) in each group were generally decreased after weighting (see Supplementary Fig. S2.1–S2.7 for details of ASMD before and after weighting). In more detail, for groups with a larger number of patients, ASMDs for many patient background factors were less than 0.1 after weighting. For groups with fewer patients, ASMDs were decreased but some of ASMDs were still over 0.1 after weighting. In the secondary outcome analysis, the trend of ASMDs was consistent with the results of the primary outcome (see Supplementary Fig. S3.1–S3.7 for more details on the secondary outcome). As shown in Table 1, the median follow-up periods and the median numbers of tests per 100 person-days were 30-166.5 days and 1 or more in all groups, respectively, except in exposure group 1 (captopril). For exposure group 1 (captopril), most patients were prescribed only once (the median follow-up period was 30 days) without liver function tests during the follow-up period. The percentage for occurrences of the primary outcome in each group was approximately 1–4%, but no occurrence was observed for exposure group 6 (quinapril hydrochloride) and exposure group 9 (benazepril hydrochloride). The median duration to occurrence of the primary outcome in each group with ≥10 outcomes was in the range of 24–245 days. The results of the secondary outcome were consistent with those of the primary outcome.

**Table 1:** Summary of follow-up periods, implementation status of tests, number of outcomes, and duration to occurrence of outcome

		Number of patients	Primary outcome^a^							Secondary outcome^b^		
			Follow-up period (days)		mplementation status of tests(/100-person days)^c^		Number of outcome (%)^d^	Duration to occurrence of outcome (days)		Number of outcome (%)^d^	Duration to occurrence of outcome (days)	
			Median	(Interquartile range)	Median	(Interquartile range)		Median	(Interquartile range)		Median	(Interquartile range)
	Control group: Enalapril maleate	12,448	92.0 (46.0–412.0)		4.0 (1.0–12.0)		404 (3.25)	70.0 (13.0–517.0)		584 (4.69)	51.0 (16.5–344.5)	
	Exposure group 1: Captopril	3242	30.0 (30.0–31.0)		0.0 (0.0–1.0)		38 (1.17)	24.0 (3.0–54.0)		43 (1.33)	32.0 (6.0–67.0)	
	Exposure group 2: Alacepril	408	59.5 (36.0–179.5)		2.0 (0.0–6.0)		10 (2.45)	25.0 (13.0–98.0)		18 (4.41)	42.5 (20.0–84.0)	
	Exposure group 3: Delapril hydrochloride	18	32.5 (9.0–38.0)		1.0 (0.0–3.0)		<10*	1.0 (*-*)		<10*	1.0 (*-*)	
	Exposure group 4: Cilazapril hydrate	128	166.5 (69.5–1088.0)		3.0 (1.0–16.5)		<10*	789.5 (*-*)		<10*	91.0 (*-*)	
	Exposure group 5: Imidapril hydrochloride	3621	79.0 (42.0–351.0)		3.0 (1.0–10.0)		90 (2.49)	35.5 (7.0–294.0)		134 (3.70)	48.5 (8.0–289.0)	
	Exposure group 6: Quinapril hydrochloride	17	30.0 (5.0–36.0)		1.0 (1.0–3.0)		0 (0.00)	(–)		0 (0.00)	(–)	
	Exposure group 7: Perindopril erbumine	5294	119.0 (53.0–368.0)		5.0 (2.0–13.0)		195 (3.68)	68.0 (11.0–325.0)		260 (4.91)	49.0 (14.0–246.5)	
	Exposure group 8: Lisinopril hydrate	3822	87.0 (46.0–364.0)		4.0 (1.0–10.0)		100 (2.62)	72.0 (15.0–424.5)		139 (3.64)	53.0 (16.0–352.0)	
	Exposure group 9: Benazepril hydrochloride	26	116.0 (44.0–596.0)		4.5 (1.0–12.0)		0 (0.00)	(–)		0 (0.00)	(–)	
	Exposure group 10: Temocapril hydrochloride	716	131.5 (44.0–626.0)		4.0 (1.0–14.0)		19 (2.65)	245.0 (10.0–874.0)		28 (3.91)	224.5 (9.5–853.0)	
	Exposure group 11: Trandolapril	77	41.0 (28.0–74.0)		3.0 (1.0–6.0)		<10*	6.0 (*-*)		<10*	6.0 (*-*)	

*When a value was <10, it was shown as an aggregated value based on the MID-NET^Ⓡ^ publication rule

*ALP* alkaline phosphatase, *ALT* alanine aminotransferase, *AST* aspartate aminotransferase, *T-Bil* total bilirubin

^a^The primary outcome was defined by following criteria based on the severity classification of the Ministry of Health, Labor and Welfare : AST, ALT, ALP and T-Bil were all grade 1 or lower (i.e., AST < 100 U/L, ALT < 100 U/L, ALP < 805 U/L, T-Bil < 3.0 mg/dL) during the 90 days before the record date of the condition such as a) both AST and ALT were grade 2 or higher on the same day (i.e., AST ≥ 100 U/L and ALT ≥ 100 U/L), or b) both ALP and T-Bil were grade 2 or higher on the same day (i.e, ALP ≥ 805 U/L and T-Bil ≥ 3.0 mg/dL)

^b^The secondary outcome was any liver dysfunction of grade 3 or higher on the Common Terminology Criteria for Adverse Events (CTCAE) v5.0

^c^Implementation status of liver function tests was judged based on the presence or absence of testing results that could be evaluated for the occurrence of outcomes (e.g., the measurement of AST and ALT on the same day or measurement of ALP and T-Bil on the same day)

^d^Representing the number and percentage of patients meeting the outcome definition during the follow-up period

**Fig. 2:**
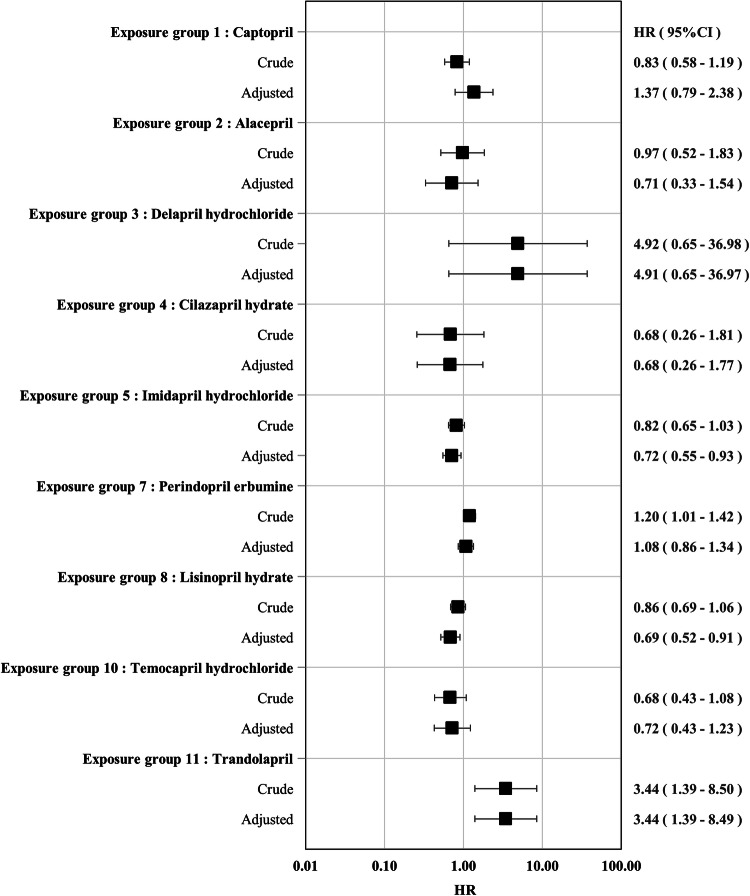
Hazard ratios and 95% confidence intervals for each exposure group compared with control group (enalapril maleate) for primary outcome. HR hazard ratio, CI confidence interval Adjusted hazard ratios were estimated using inverse probability weighting based on the high-dimensional propensity scores calculated by logistic regression for each combination between the control and exposure groups. The estimation accuracy of the adjusted hazard ratio was low for groups with a small number of patients

The HRs and their 95% CIs for the primary outcome are presented in Fig. 2. The aHRs for many exposure groups compared with the control group (enalapril maleate) were in the range of 0.68–1.37 with the 95% CI including 1.00 (exposure group 1 (captopril), 1.37(0.79–2.38); exposure group 2 (alacepril), 0.71(0.33–1.54); exposure group 4 (cilazapril hydrate), 0.68(0.26–1.77); exposure group 7 (perindopril erbumine), 1.08(0.86–1.34); and exposure group 10 (temocapril hydrochloride), 0.72(0.43–1.23)). The aHRs for exposure group 5 (imidapril hydrochloride) and exposure group 8 (lisinopril hydrate) were 0.72 (0.55–0.93) and 0.69 (0.52–0.91), respectively, with the upper limit of the CI < 1.00. For exposure group 3 (delapril hydrochloride), the aHR was 4.91 (0.65–36.97) but had a large CI including 1.00. For exposure group 11 (trandolapril), the aHR was 3.44(1.39–8.49), with the lower limit of the CI > 1.00. All sensitivity analyses for the primary outcome also showed similar trends to those of the primary analysis (see Supplementary Fig. S4 for aHRs in the sensitivity analyses).

**Fig. 3:**
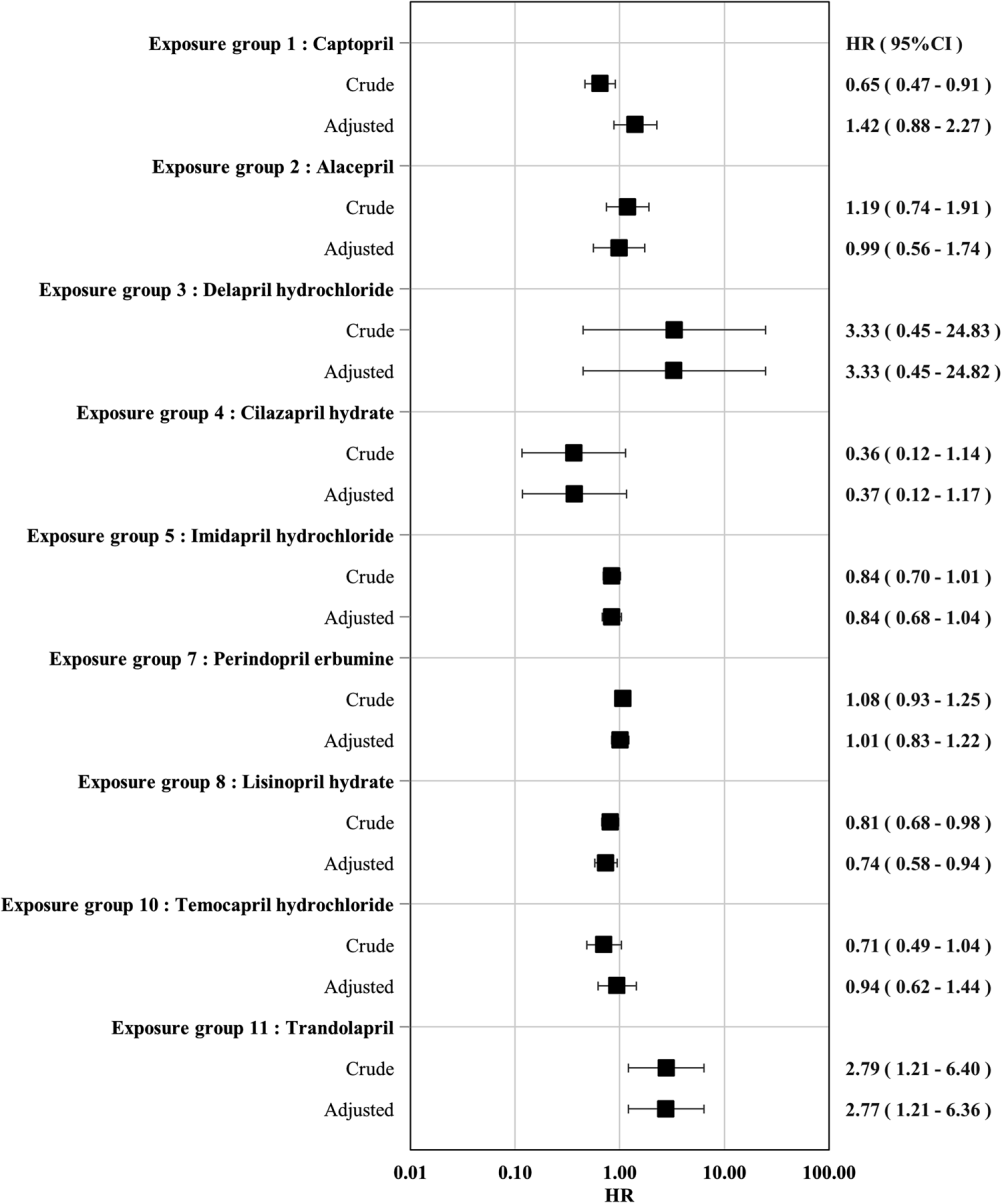
Hazard ratios and 95% confidence intervals for each exposure group compared with control group (enalapril maleate) for secondary outcome. HR hazard ratio, CI confidence interval Adjusted hazard ratios were estimated using inverse probability weighting based on the high-dimensional propensity scores calculated by logistic regression for each combination between the control and exposure groups. The estimation accuracy of the adjusted hazard ratio was low for groups with a small number of patients

The aHRs and their 95% CIs for the secondary outcome are presented in Fig. 3. The results were generally consistent with the primary outcome analysis except the following: aHR for exposure group 5 (imidapril hydrochloride) compared with the control group (enalapril maleate) was still less than 1.0 (aHR(95%CI), 0.84(0.68–1.04)) but its 95% CI included 1.00. All sensitivity analysis for the secondary outcome also showed similar trends to those of the primary analysis targeted on the secondary outcome (see Supplementary Fig. S5 for aHRs in the sensitivity analyses).

Regarding the disproportionality analysis based on ICSRs, ICSRs with SMQ “Drug-related hepatic disorders-comprehensive search” with targeted ACE inhibitors in this study were identified in the overall dataset as shown in Table 2. The IC for captopril was higher with statistical significance (IC = 0.1, IC_0.25_ = 0.05) in the overall dataset but not the Japan subset. For other ACE inhibitors, no significant differences were observed, or no cases were identified in the overall dataset and/or Japan subset.

**Table 2:** Information component values for drug related liver dysfunction in the VigiBase dataset

Active ingredient	Overall dataset ^a^					Japan subset ^b^				
	Number of	Number of ICSRs of drug related liver dysfunction		IC	IC_0.25_	Number of	Number of ICSRs of drug		IC	IC_0.25_
	ICSRs					ICSRs	related liver dysfunction			
	(Observed)	Observed	Expected			(Observed)	Observed	Expected		
Exposure group 1: Captopril	32,054	846	763	0.1	0.05	134	6	9	−0.6	−2.0
Exposure group 2: Alacepril	133	0	—	—	—	128	0	—	—	—
Exposure group 3: Delapril hydrochloride	88	2	2	−0.1	−2.6	22	0	—	—	—
Exposure group 5: Imidapril hydrochloride	478	19	11	0.7	−0.007	201	16	14	0.2	−0.6
Exposure group 7: Perindopril erbumine	29,672	425	706	−0.7	−0.9	54	2	4	−3.4	−0.8

ICSRs, Individual Case Safety Reports; IC, Information Component; IC_0.25_, lower limit of the 95% confidence interval of IC

^a^The number of ISCRs of drug related liver dysfunction reported for all drugs was 902,658 in the overall dataset

^b^The number of ISCRs of drug related liver dysfunction reported for all drugs was 31,442 in the Japan subset

## Discussion

The risk of liver dysfunction observed in this study was inconsistent among ACE inhibitors in comparison with enalapril maleate in both primary and secondary outcomes. Namely, the 95% CIs of aHR for many ACE inhibitors included 1.0, but increased or decreased aHR was observed for exposure group 5 (imidapril hydrochloride), exposure group 8 (lisinopril hydrate), and exposure group 11 (trandolapril), and no cases were identified in exposure group 6 (quinapril hydrochloride) and exposure group 9 (benazepril hydrochloride) in the primary outcome analysis. The incidence proportions of liver dysfunction for each ACE inhibitor in this study were approximately 1–5%, which are relatively higher than those reported in previous studies (0.2–2%) [26, 27]. The differences may be due to differences in outcome definitions and study designs. Specifically, identifying liver dysfunction based on laboratory test values in this study may allow for more objective detection of events, including those typically not captured in questionnaire-based surveillance, such as events disconfirmed by physicians.

These results showed that the risk of liver dysfunction with ACE inhibitors varies among individual drugs. This consideration could be supported by data in LiverTox database which provides likelihood scores of individual drugs for drug-induced liver toxicity based on clinical reports and FDA documents, showing the different risk among ACE inhibitors (e.g., E* [unproven but suspected]: perindopril, quinapril, trandolapril; D [possible]: benazepril; B [highly likely]: captopril, enalapril, lisinopril) [26]. Another study using RWD also showed differences in the incidence rate of liver impairment with ACE inhibitors (e.g., 5.8/10,000 PY(person-years) for captopril, 1.9/10,000 PY for enalapril, and 0.97/10,000 PY for lisinopril) [28]. These suggests that the risk of liver dysfunction with ACE inhibitors is unlikely to be a class-effect.

It should be noted that the 95% CIs including 1.0 observed for many ACE inhibitors compared to enalapril maleate may imply the risk of liver dysfunction because the PI of enalapril maleate includes the liver dysfunction warning as CSAR. However, the results should be carefully interpreted because no significant differences on the risk of liver dysfunction between enalapril maleate and placebo have been reported [26]. Furthermore, the disproportionality analysis based on ICSRs targeted for alacepril, delapril hydrochloride, imidapril hydrochloride, and perindopril erbumine indicates no signals of liver dysfunction in both the overall dataset and Japan subset. In taking all results into consideration, it suggests that no additional safety measures are necessary at present for ACE inhibitors that do not carry a liver dysfunction-related warning as CSAR in Japan.

Regarding exposure group 1 (captopril), most patients were prescribed only once without liver function tests during the follow-up period, and the follow-up period is short, indicating that this drug was likely prescribed for primary diagnosis of aldosteronism rather than treatment for hypertension. Thus, the aHR for this drug should be carefully interpreted. Moreover, a signal of liver dysfunction for captopril was identified in the disproportionality analysis based on ICSRs in the overall dataset, although the signal was weak and insubstantial due to the actual IC value being 0.1. When checking domestic ICSRs reported to the PMDA since 2004, 3 cases meeting the MedDRA ver.26.1 SMQ “Drug-induced liver injury—Comprehensive search” criteria were identified and were classified as grade 3 or higher based on the CTCAE v5.0, including 1 case with suspected causality. However, only a limited number of ICSRs for captopril with suspected causality have been reported considering a long history of marketing of this drug in Japan since February 1983. These results based on the database study, the disproportionality analysis, and the causal assessment for ICSRs suggest a potential risk of liver dysfunction with captopril but more evidence is necessary for making a conclusion as to whether or not additional safety measures for this drug are necessary.

This study has several strengths. First, the study used laboratory test results of AST, ALT, ALP, and T-Bil as an outcome of liver dysfunction from the MID-NET^®^, a reliable database [9]. Second, the use of HDPS methodology allowed for extensive adjustment of confounding factors. Although it is recommended to carefully assess confounding factors in advance using a directed acyclic graph, it can be challenging to comprehensively identify all factors at the planning stage, especially for outcomes involving many variables, such as liver dysfunction. As the usefulness of HDPS for examining outcomes with unclear contributing factors has been reported [29, 30] and different factors were extracted for each cohort by HDPS in this study (Supplementary Fig. S2 and Supplementary Fig. S3), the study provides a real-world example applying HDPS in drug safety assessment, which highlights the importance of assessing the validity of results by accounting for variables other than those predefined. In contrast, the following limitations should be considered when evaluating the study results. First, since the HDPS estimation was performed for each pair of exposure and control groups, direct comparisons of the results among different exposure groups might not be appropriate. Therefore, the HRs estimated in this study for each exposure group do not necessarily indicate the relative risk of liver dysfunction among exposure groups. Second, the accuracy of aHRs for groups with a small number of patients (especially delapril hydrochloride, cilazapril hydrate, and trandolapril) might be low and the results should be interpreted with caution.

### Perspective of Asia

Effective management of hypertension in Asian populations is particularly important, as the association between blood pressure and both fatal and nonfatal stroke is stronger in Asian populations who tend to exhibit higher systolic, diastolic, and mean arterial blood pressure levels than European populations [31]. Although ethnic factors on drug responses should be taken into consideration, the finding of increased risk of liver dysfunction with ACE inhibitors in Japanese patients provide valuable insights into their safety profile and appropriate clinical use.

## Conclusion

This study suggests that the risk of liver dysfunction with ACE inhibitors is unlikely to be a class-effect. Although continuous safety monitoring is necessary for promoting proper use of ACE inhibitors, the results indicate that no additional safety measures are currently required for ACE inhibitors that do not carry a liver dysfunction-related warning in Japan.

## Supplementary information

Supplementary Table S1

Supplementary Figures
