# Supplementary material for: Risk of liver dysfunction with ACE inhibitors based on real-world data from the MID-NET® in Japan
Source: Hypertens Res. 2025 Oct 10;48(12):3080–90. doi: 10.1038/s41440-025-02390-x (PMC12678171; doi:10.1038/s41440-025-02390-x)
Supplement: Supplementary file 1 — Supplementary Table S1 [file 41440_2025_2390_MOESM1_ESM.pdf]

Supplementary Table S1. Patient background

	Control group: Enalapril maleate	Exposure group 1: Captopril	Exposure group 2: Alacepril	Exposure group 3: Delapril hydrochloride	Exposure group 4: Cilazapril hydrate	Exposure group 5: Imidapril hydrochloride	Exposure group 6: Quinapril hydrochloride	Exposure group 7: Perindopril erbumine	Exposure group 8: Lisinopril hydrate	Exposure group 9: Benazepril hydrochloride	Exposure group 10: Temocapril hydrochloride	Exposure group 11: Trandolapril
n	12,448 (100.00)	3,242 (100.00)	408 (100.00)	18 (100.00)	128 (100.00)	3,621 (100.00)	17 (100.00)	5,294 (100.00)	3,822 (100.00)	26 (100.00)	716 (100.00)	77 (100.00)
Sex												
Male	7,552 (60.67)	1,361 (41.98)	215 (52.70)	<10* (*)	74 (57.81)	2,189 (60.45)	<20* (*)	3,288 (62.11)	2,291 (59.94)	14 (53.85)	419 (58.52)	38 (49.35)
Female	4,896 (39.33)	1,881 (58.02)	193 (47.30)	<20* (*)	54 (42.19)	1,432 (39.55)	<10* (*)	2,006 (37.89)	1,531 (40.06)	12 (46.15)	297 (41.48)	39 (50.65)
Age												
<65	4,312 (34.64)	1,862 (57.43)	70 (17.16)	0 (0.00)	70 (54.69)	1,094 (30.21)	<10* (*)	1,352 (25.54)	985 (25.77)	13 (50.00)	346 (48.32)	17 (22.08)
65≤	8,136 (65.36)	1,380 (42.57)	338 (82.84)	18 (100.00)	58 (45.31)	2,527 (69.79)	<20* (*)	3,942 (74.46)	2,837 (74.23)	13 (50.00)	370 (51.68)	60 (77.92)
Baseline liver function												
Normal	10,124 (81.33)	2,862 (88.28)	366 (89.71)	<20* (*)	118 (92.19)	3,115 (86.03)	<20* (*)	4,335 (81.89)	3,108 (81.32)	<30* (*)	634 (88.55)	61 (79.22)
Grade 1	2,324 (18.67)	380 (11.72)	42 (10.29)	<10* (*)	10 (7.81)	506 (13.97)	<10* (*)	959 (18.11)	714 (18.68)	<10* (*)	82 (11.45)	16 (20.78)
history of liver function test abnormal												
Normal (including missing)	8,758 (70.36)	2,658 (81.99)	331 (81.13)	13 (72.22)	117 (91.41)	2,818 (77.82)	13 (76.47)	3,718 (70.23)	2,721 (71.19)	24 (92.31)	586 (81.84)	55 (71.43)
Grade 1	2,729 (21.92)	504 (15.55)	65 (15.93)	<10* (*)	11 (8.59)	660 (18.23)	<10* (*)	1,238 (23.38)	873 (22.84)	<10* (*)	109 (15.22)	<20* (*)
Grade 2 ≤	961 (7.72)	80 (2.47)	12 (2.94)	<10* (*)	0 (0.00)	143 (3.95)	<10* (*)	338 (6.38)	228 (5.97)	<10* (*)	21 (2.93)	<10* (*)
Comorbidities												
Prescriptions for diabetes medications	3,226 (25.92)	452 (13.94)	93 (22.79)	<10* (*)	12 (9.38)	1,167 (32.23)	<10* (*)	1,341 (25.33)	878 (22.97)	<10* (*)	206 (28.77)	21 (27.27)
Prescriptions for dyslipidemia medications	4,896 (39.33)	651 (20.08)	157 (38.48)	<10* (*)	38 (29.69)	1,496 (41.31)	10 (58.82)	2,454 (46.35)	1,347 (35.24)	10 (38.46)	270 (37.71)	32 (41.56)
Comorbid heart failure	2,847 (22.87)	194 (5.98)	55 (13.48)	<10* (*)	0 (0.00)	354 (9.78)	<10* (*)	1,679 (31.72)	966 (25.27)	<10* (*)	51 (7.12)	10 (12.99)

* When a value was < 10, it was shown as an aggregated value based on the MID-NET® publication rule.
