# Supplementary material for: Risk of liver dysfunction with ACE inhibitors based on real-world data from the MID-NET® in Japan
Source: Hypertens Res. 2025 Oct 10;48(12):3080–90. doi: 10.1038/s41440-025-02390-x (PMC12678171; doi:10.1038/s41440-025-02390-x)
Supplement: Supplementary file 2 — Supplementary Figures [file 41440_2025_2390_MOESM2_ESM.pdf]

Supplementary Figure S1. Design diagram

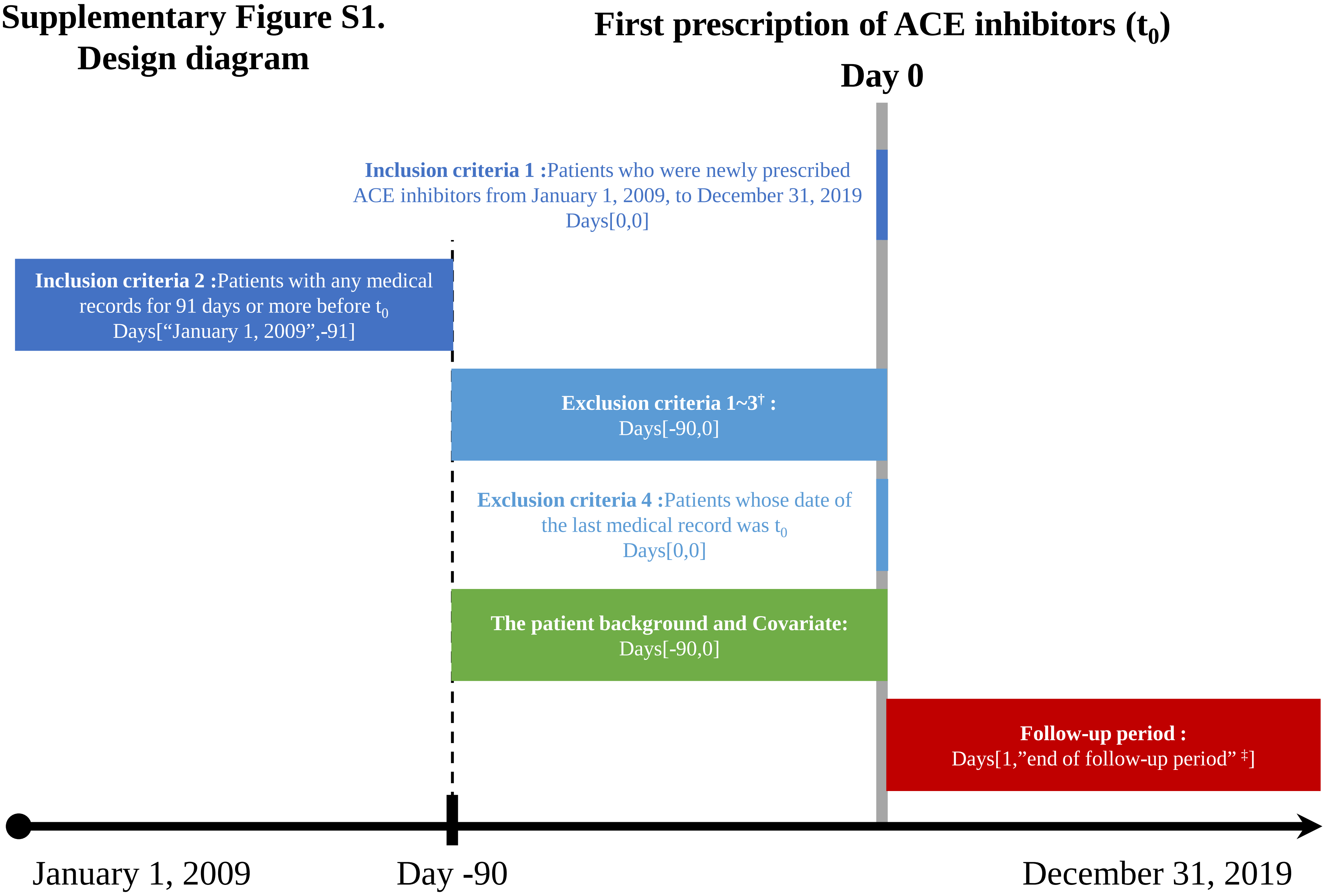

† Exclusion criteria 1~3 were as outlined in the following criteria : (1) patients with liver dysfunction defined as grade 2 or higher (aspartate aminotransferase ≥ 100 U/L, alanine aminotransferase ≥ 100 U/L, alkaline phosphatase ≥ 805 U/L, total bilirubin ≥ 3.0 mg/dL), (2) patients prescribed anticancer drugs or who underwent radiation therapy, (3) patients prescribed antiviral drugs for hepatitis B or C.

‡ The end of follow-up was the earliest date among the following: (1) 30 days after the end of the treatment period, (2) the start date of a different ACE inhibitor prescription, or (3) the date of the last medical record for a patient during the study period.

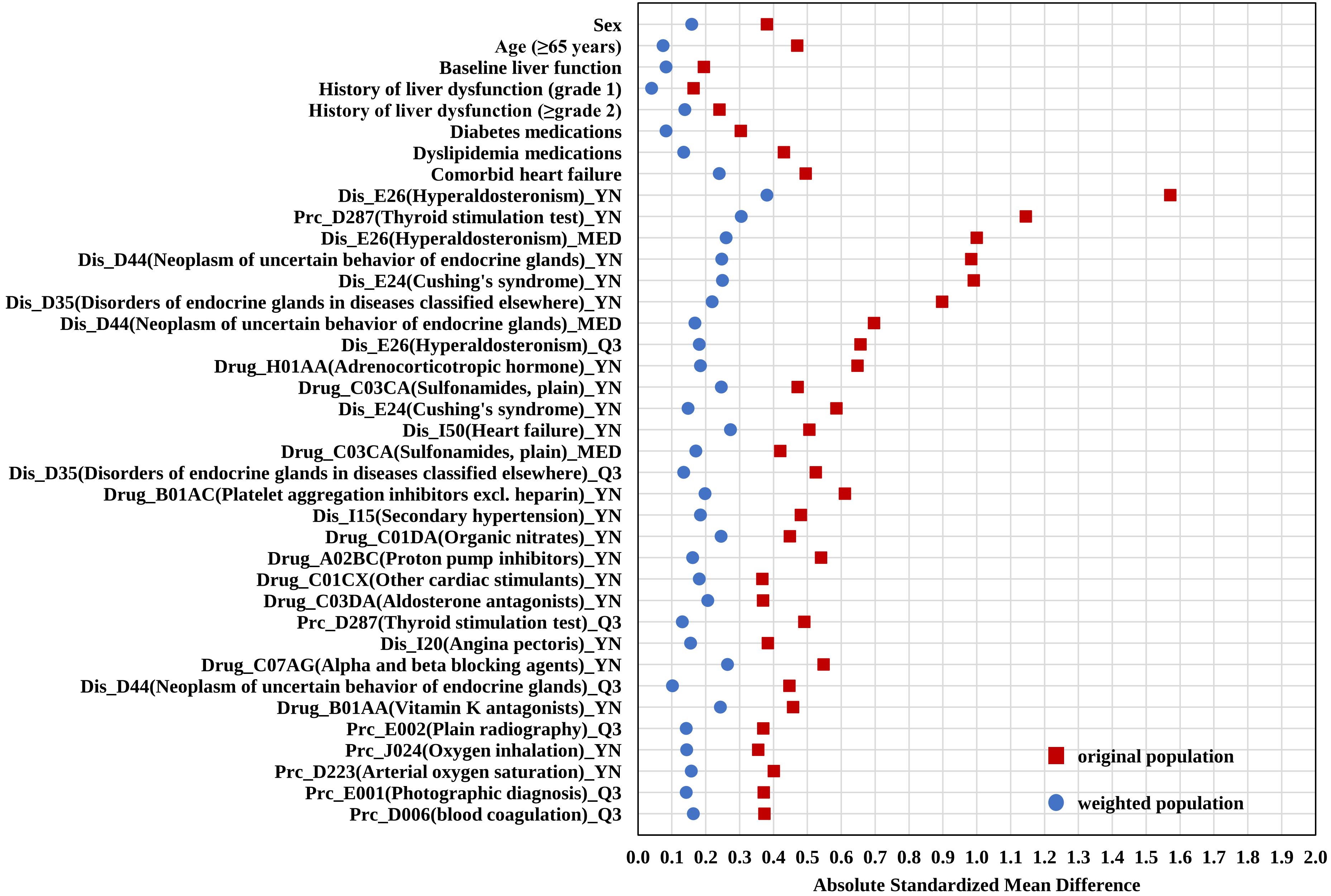

Supplemental Figure S2.1 Absolute standardized mean difference for basic covariates and top 30 covariates based on high-dimensional propensity score method comparing Exposure group 1 (Captopril) to Control group (Enalapril maleate) in the original and the weighted population for the main outcome.

Dis, Disease; Prc, Procedure; YN, variable type (Yes or No); MED, variable type (<Median, Median≤); Q3, variable type (<Q3, Q3≤)

*The top 30 covariates are represented by "the dimension_code(description)_variable type".

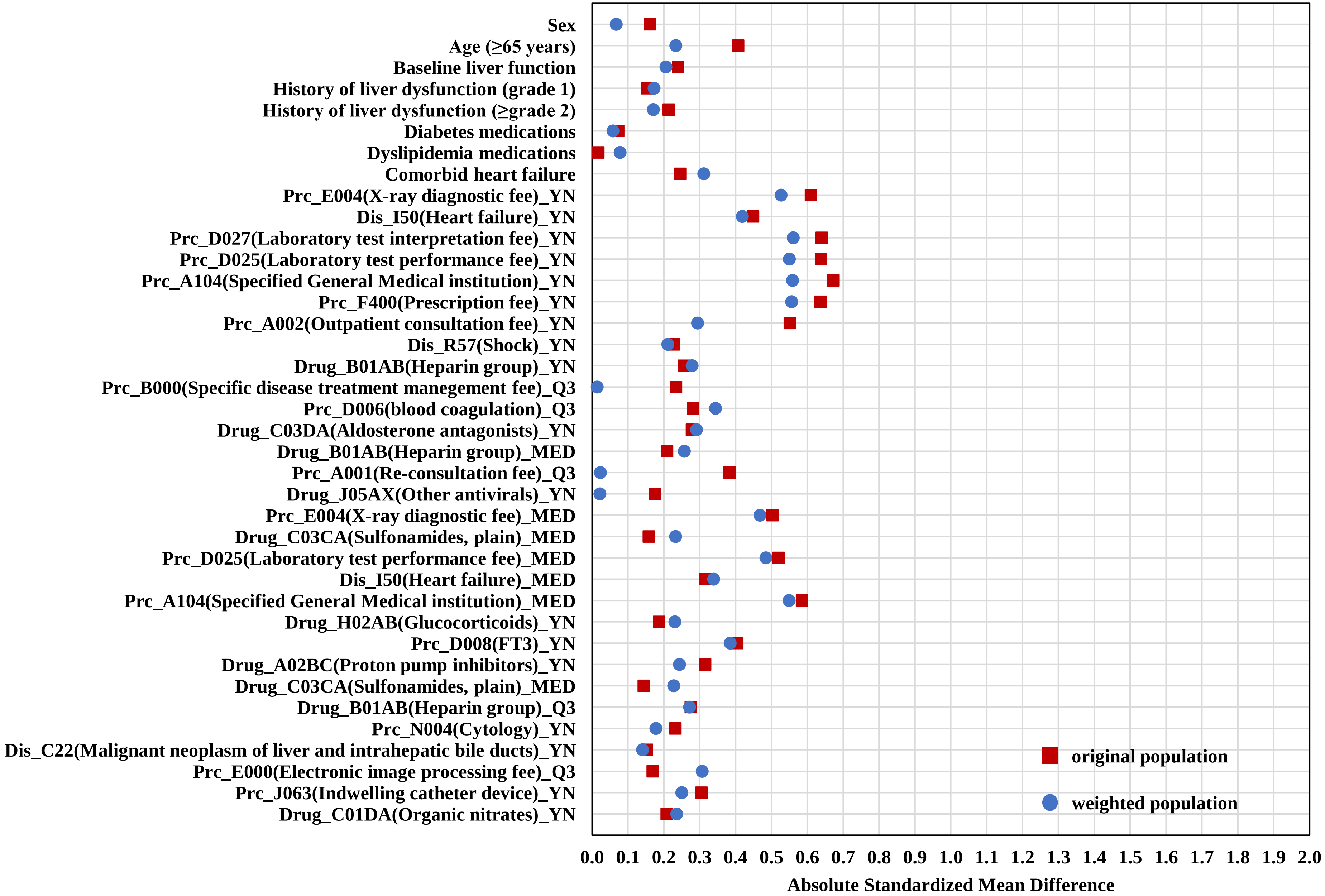

Supplemental Figure S2.2 Absolute standardized mean difference for basic covariates and top 30 covariates based on high-dimensional propensity score method comparing Exposure group 2 (Alacepril) to Control group (Enalapril maleate) in the original and the weighted population for the main outcome.

Dis, Disease; Prc, Procedure; YN, variable type (Yes or No); MED, variable type (<Median, Median≤); Q3, variable type (<Q3, Q3≤)

*The top 30 covariates are represented by "the dimension_code(description)_variable type".

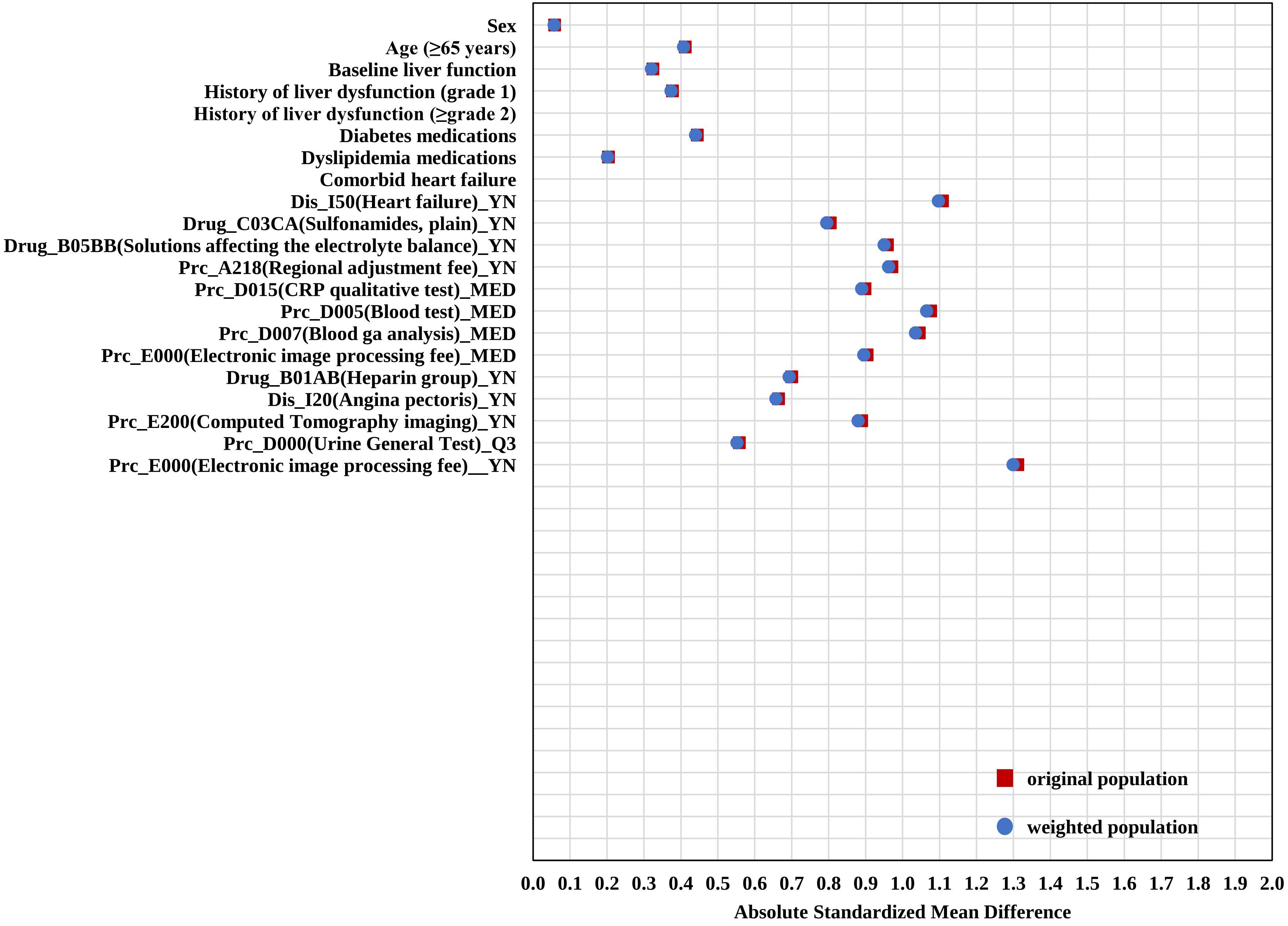

Supplemental Figure S2.3 Absolute standardized mean difference for basic covariates and top 30 covariates based on high-dimensional propensity score method comparing Exposure group 3 (Cilazapril hydrate) to Control group (Enalapril maleate) in the original and the weighted population for the main outcome.

Dis, Disease; Prc, Procedure; YN, variable type (Yes or No); MED, variable type (<Median, Median≤); Q3, variable type (<Q3, Q3≤)

*The top 30 covariates are represented by "the dimension_code(description)_variable type".

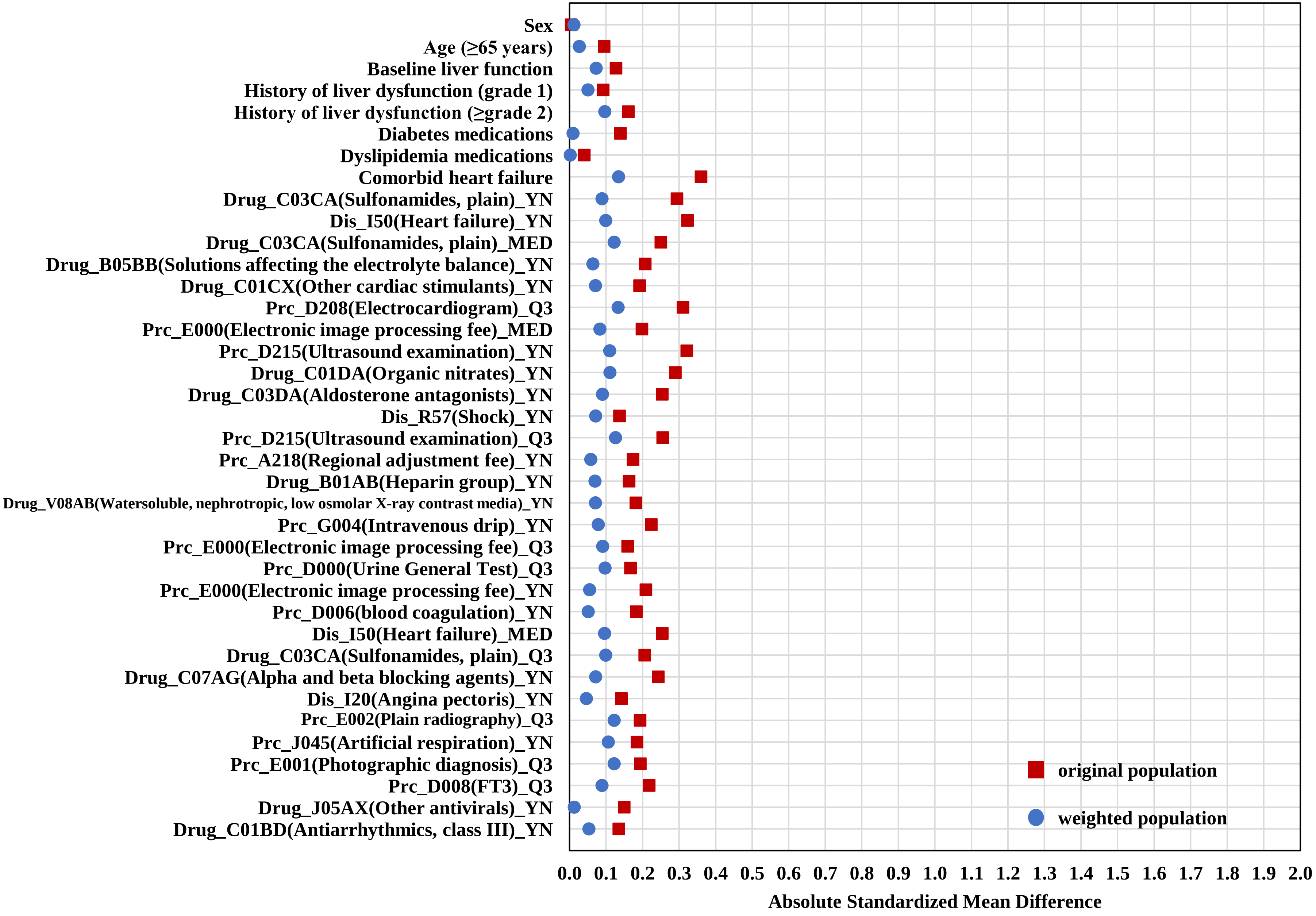

Supplemental Figure S2.4 Absolute standardized mean difference for basic covariates and top 30 covariates based on high-dimensional propensity score method comparing Exposure group 5 (Imidapril hydrochlorid) to Control group (Enalapril maleate) in the original and the weighted population for the main outcome.

Dis, Disease; Prc, Procedure; YN, variable type (Yes or No); MED, variable type (<Median, Median≤); Q3, variable type (<Q3, Q3≤)

*The top 30 covariates are represented by “the dimension_code(description)_variable type”.

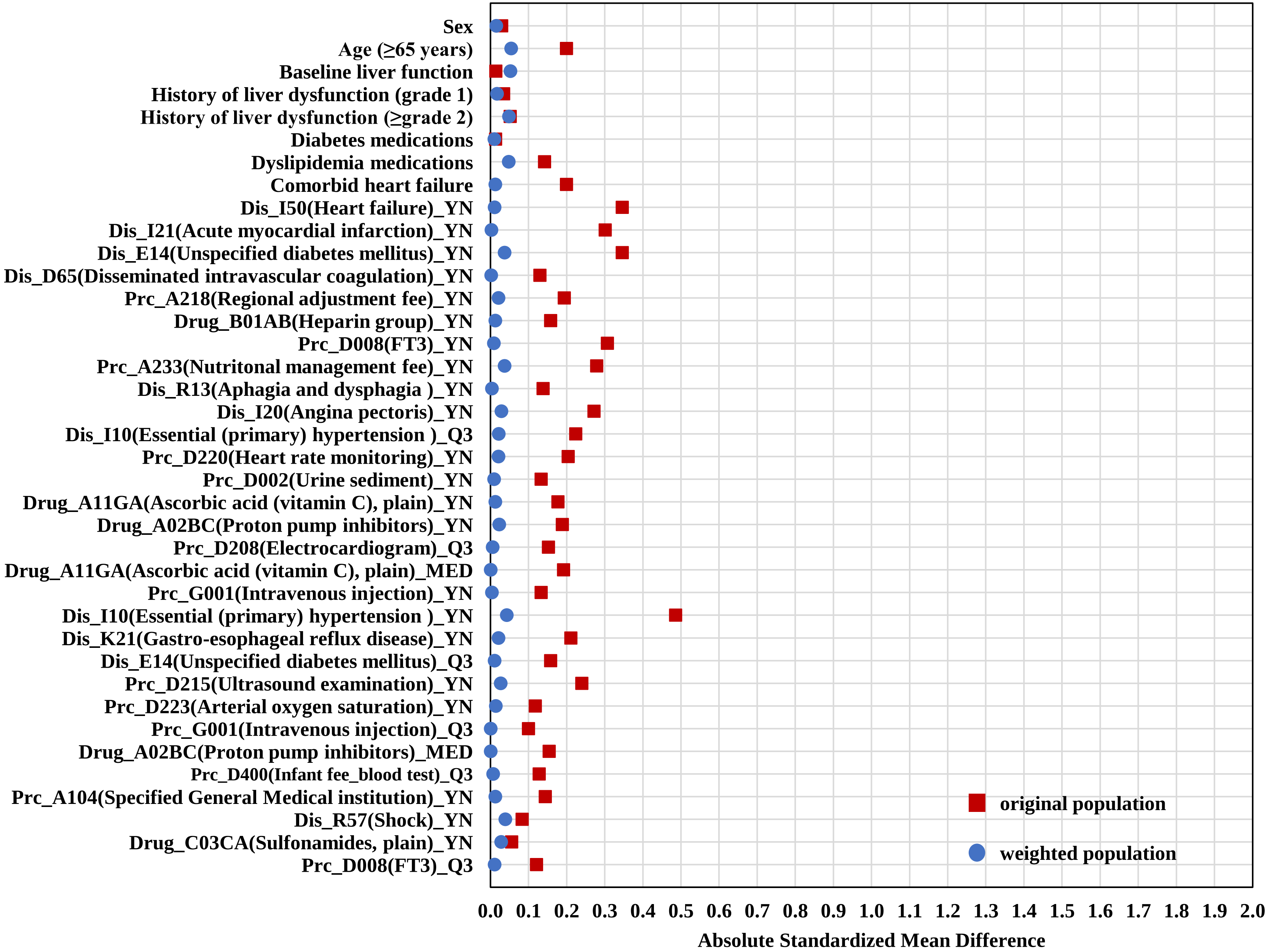

Supplemental Figure S2.5 Absolute standardized mean difference for basic covariates and top 30 covariates based on high-dimensional propensity score method comparing Exposure group 7 (Perindopril erbumine) to Control group (Enalapril maleate) in the original and the weighted population for the main outcome.
Dis, Disease; Prc, Procedure; YN, variable type (Yes or No); MED, variable type (<Median, Median≤); Q3, variable type (<Q3, Q3≤)
*The top 30 covariates are represented by "the dimension_code(description)_variable type".

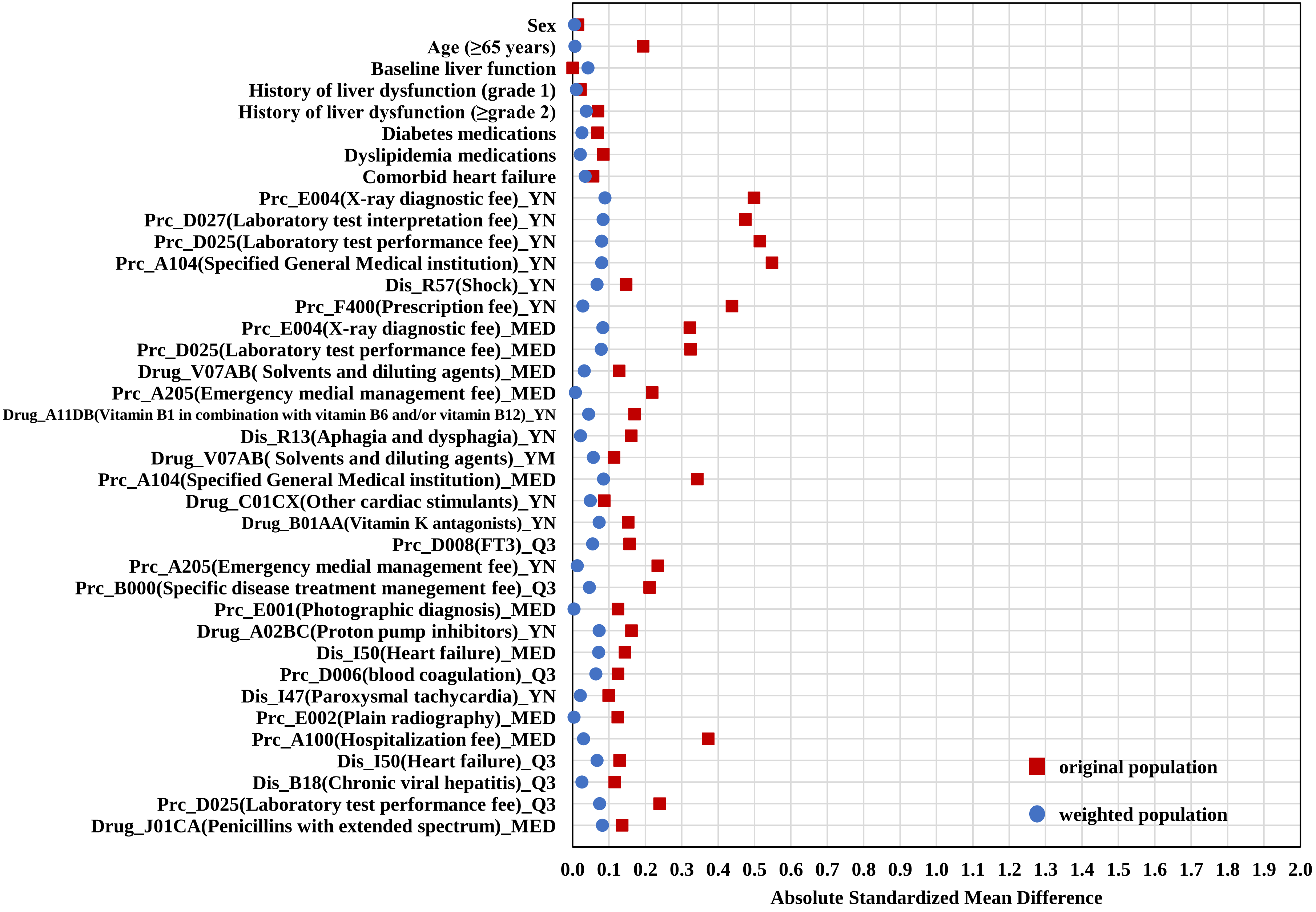

Supplemental Figure S2.6 Absolute standardized mean difference for basic covariates and top 30 covariates based on high-dimensional propensity score method comparing Exposure group 8 (Lisinopril hydrate) to Control group (Enalapril maleate) in the original and the weighted population for the main outcome.

Dis, Disease; Prc, Procedure; YN, variable type (Yes or No); MED, variable type (<Median, Median≤); Q3, variable type (<Q3, Q3≤)

*The top 30 covariates are represented by “the dimension_code(description)_variable type”.

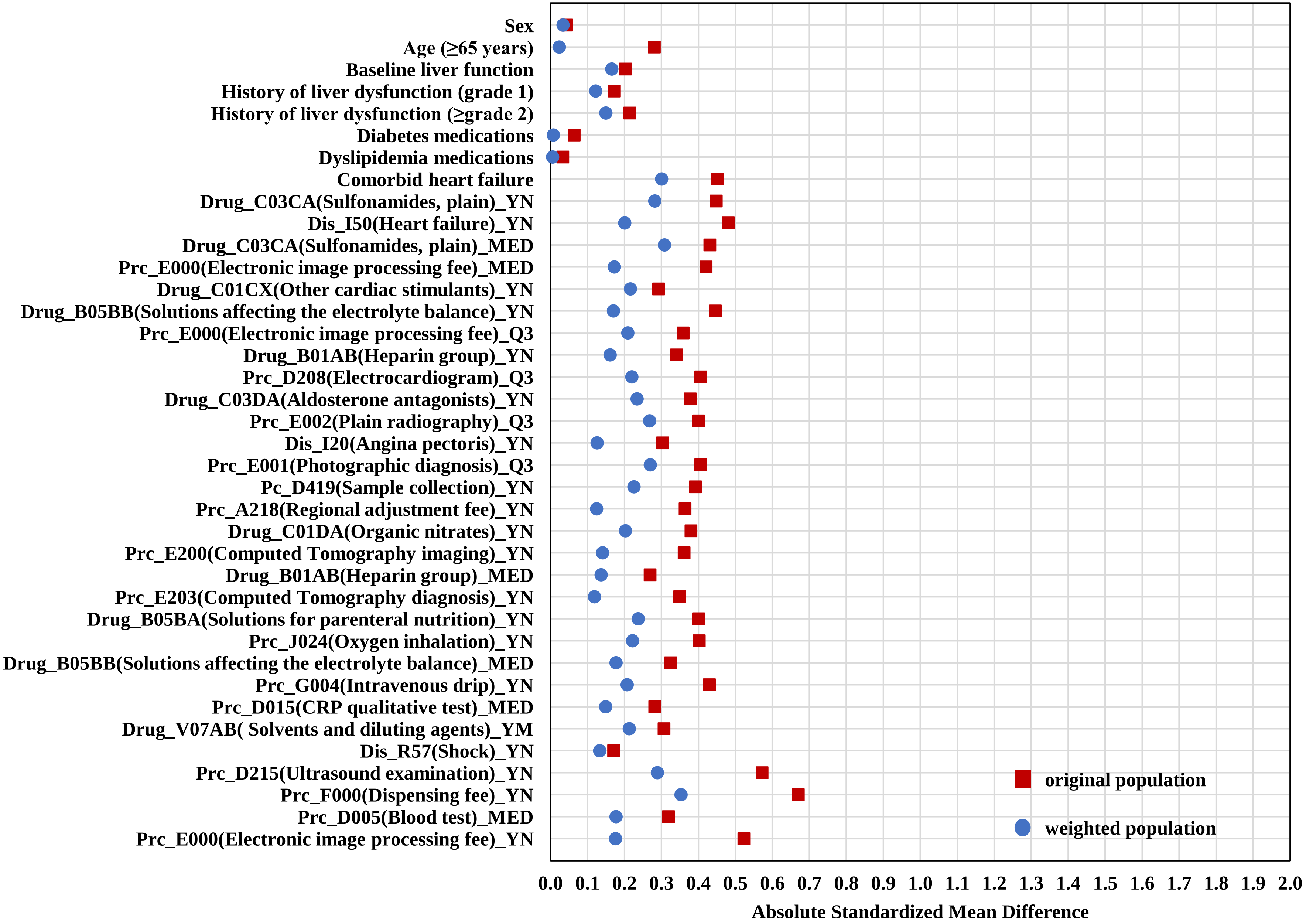

Supplemental Figure S2.7 Absolute standardized mean difference for basic covariates and top 30 covariates based on high-dimensional propensity score method comparing Exposure group 10 (Temocapril hydrochlori) to Control group (Enalapril maleate) in the original and the weighted population for the main outcome.
Dis, Disease; Prc, Procedure; YN, variable type (Yes or No); MED, variable type (<Median, Median≤); Q3, variable type (<Q3, Q3≤)
*The top 30 covariates are represented by "the dimension_code(description)_variable type".

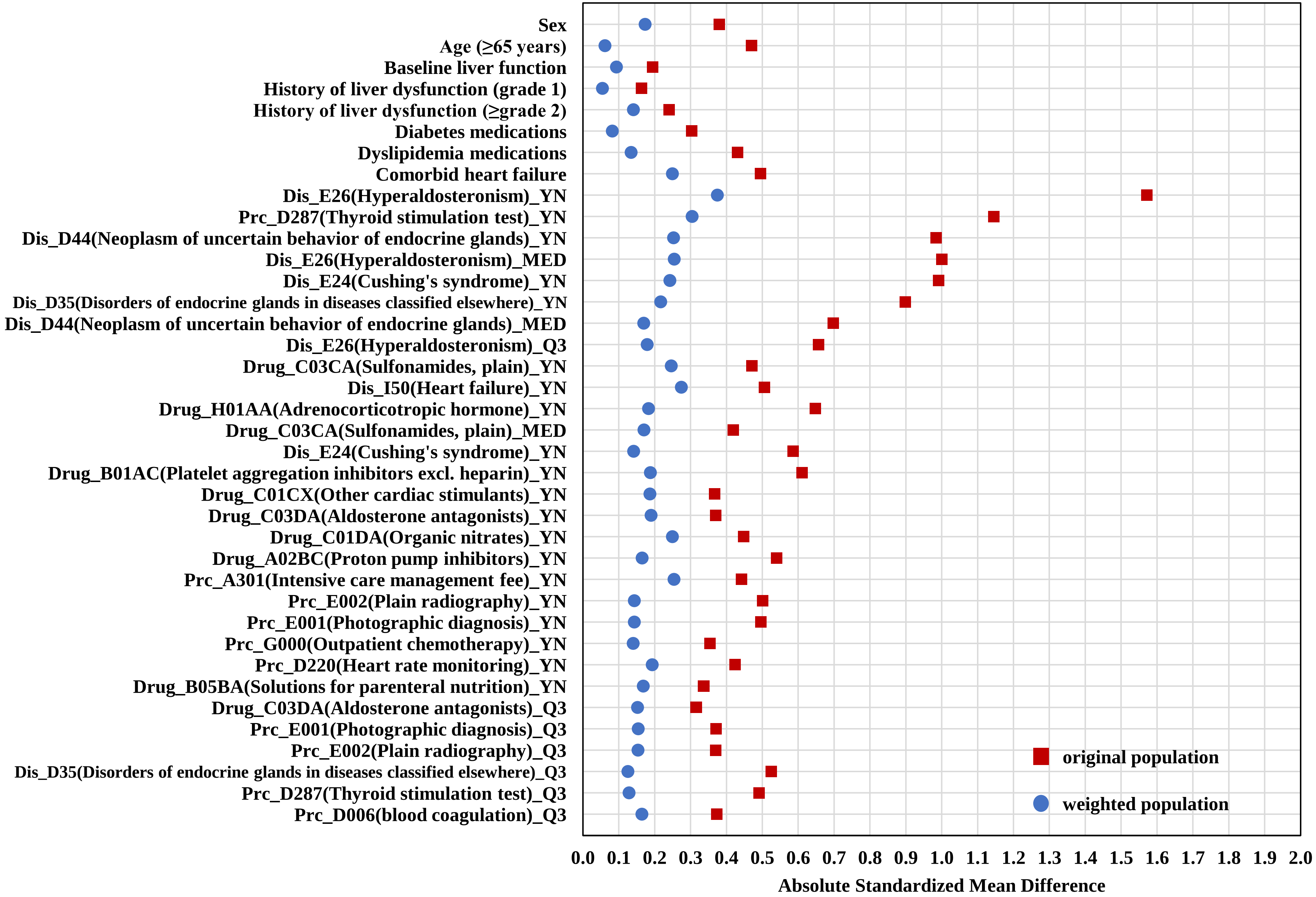

Supplemental Figure S3.1 Absolute standardized mean difference for basic covariates and top 30 covariates based on high-dimensional propensity score method comparing Exposure group 1 (Captpril) to Control group (Enalapril maleate) in the original and the weighted population for the secondary outcome.

Dis, Disease; Prc, Procedure; YN, variable type (Yes or No); MED, variable type (<Median, Median≤); Q3, variable type (<Q3, Q3≤)

*The top 30 covariates are represented by "the dimension_code(description)_variable type".

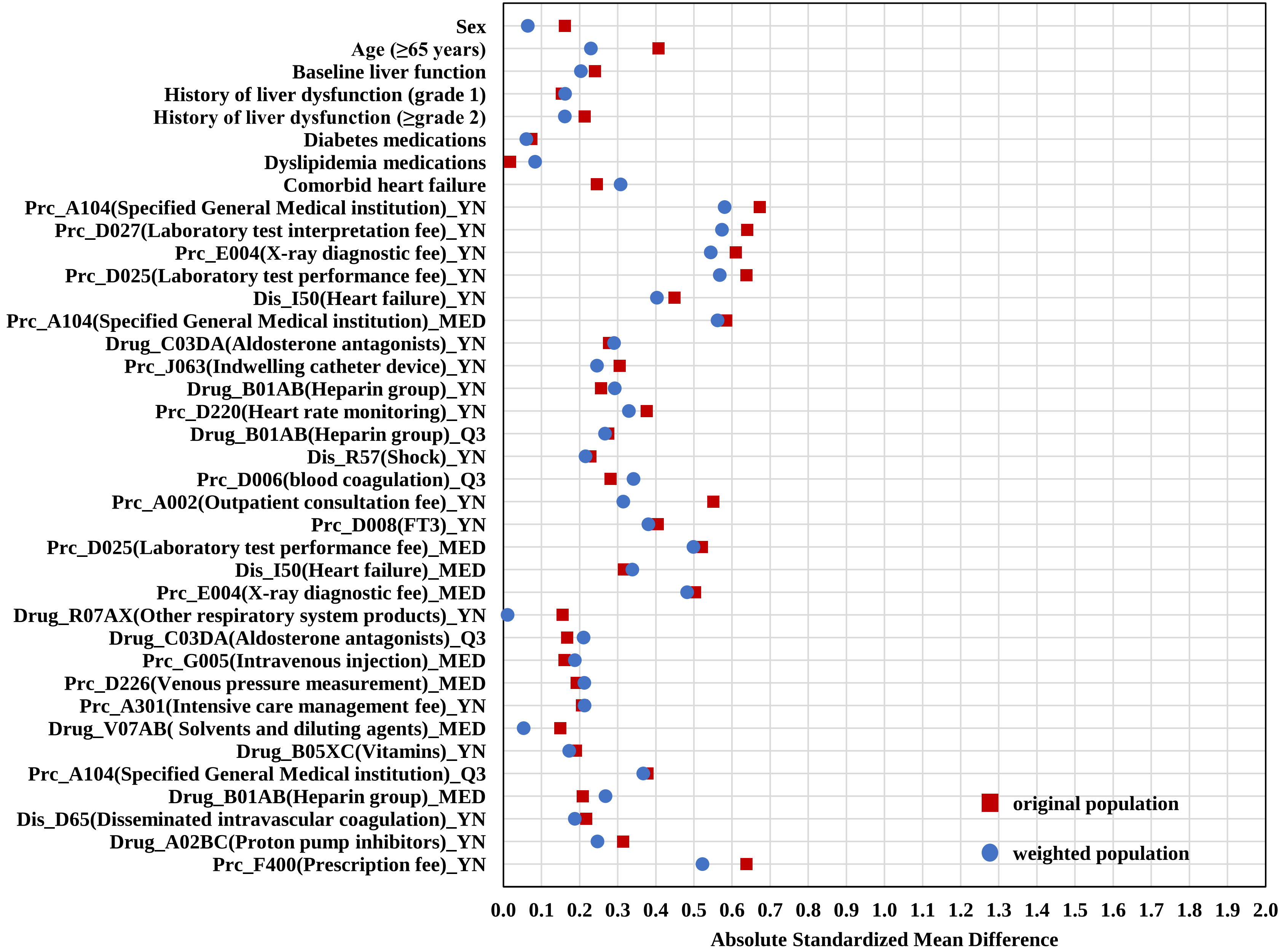

Supplemental Figure S3.2 Absolute standardized mean difference for basic covariates and top 30 covariates based on high-dimensional propensity score method comparing Exposure group 2 (Alacepril) to Control group (Enalapril maleate) in the original and the weighted population for the secondary outcome.

Dis, Disease; Prc, Procedure; YN, variable type (Yes or No); MED, variable type (<Median, Median≤); Q3, variable type (<Q3, Q3≤)

*The top 30 covariates are represented by “the dimension_code(description)_variable type”.

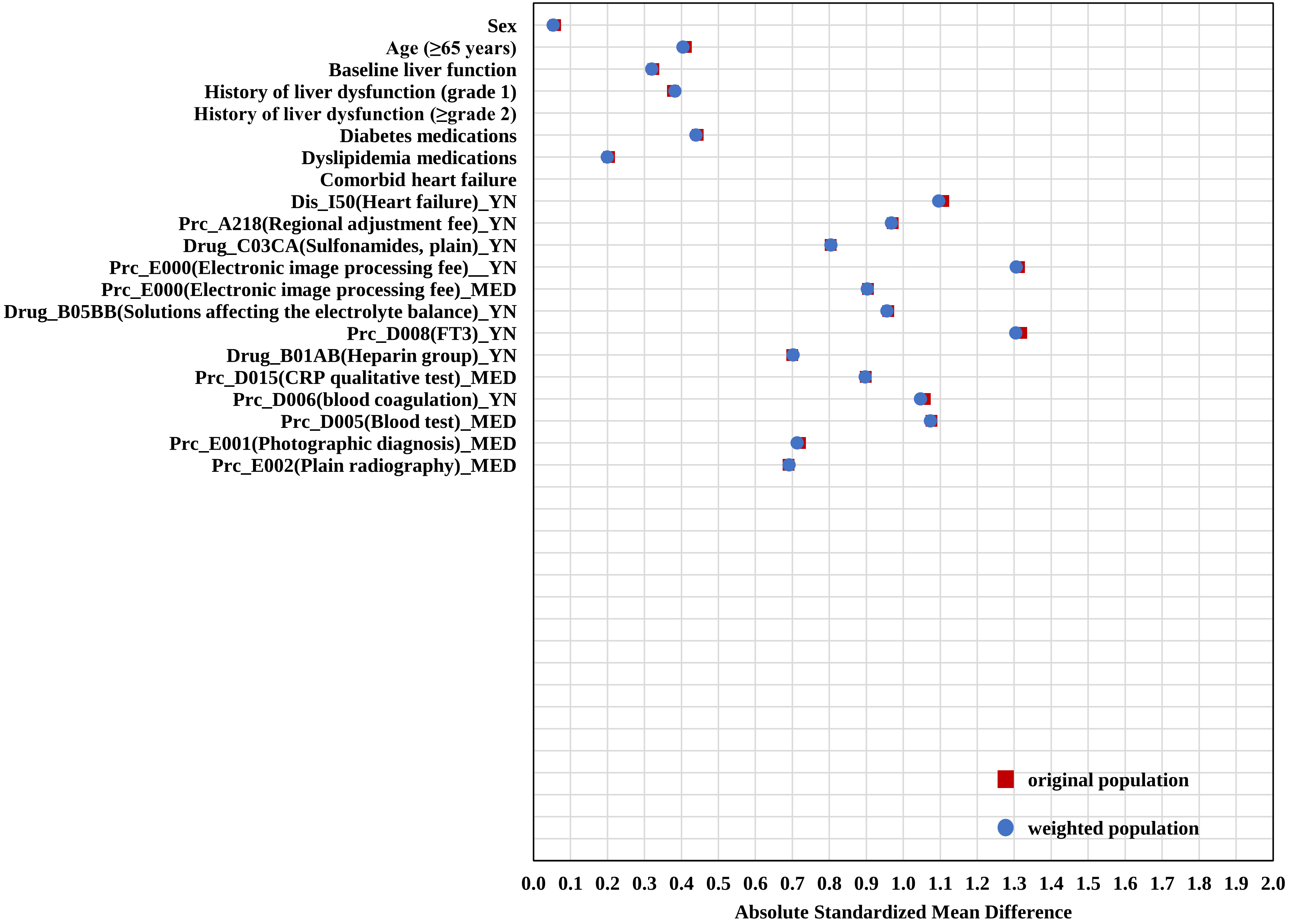

Supplemental Figure S3.3 Absolute standardized mean difference for basic covariates and top 30 covariates based on high-dimensional propensity score method comparing Exposure group 3 (Cilazapril hydrate) to Control group (Enalapril maleate) in the original and the weighted population for the secondary outcome.

Dis, Disease; Prc, Procedure; YN, variable type (Yes or No); MED, variable type (<Median, Median≤); Q3, variable type (<Q3, Q3≤)

*The top 30 covariates are represented by "the dimension_code(description)_variable type".

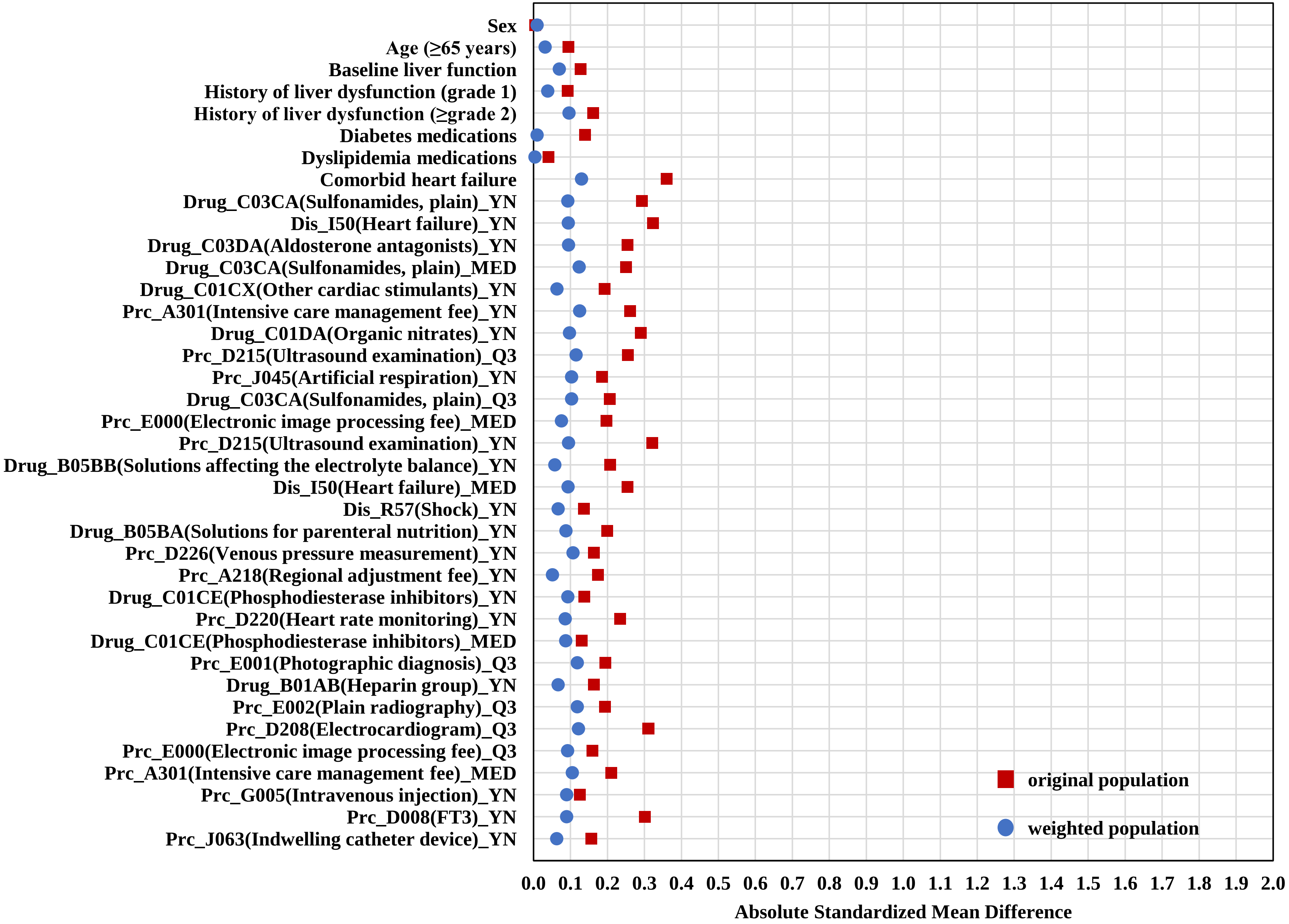

Supplemental Figure S3.4 Absolute standardized mean difference for basic covariates and top 30 covariates based on high-dimensional propensity score method comparing Exposure group 5 (Imidapril hydrochlorid) to Control group (Enalapril maleate) in the original and the weighted population for the secondary outcome.

Dis, Disease; Prc, Procedure; YN, variable type (Yes or No); MED, variable type (<Median, Median≤); Q3, variable type (<Q3, Q3≤)

*The top 30 covariates are represented by "the dimension_code(description)_variable type".

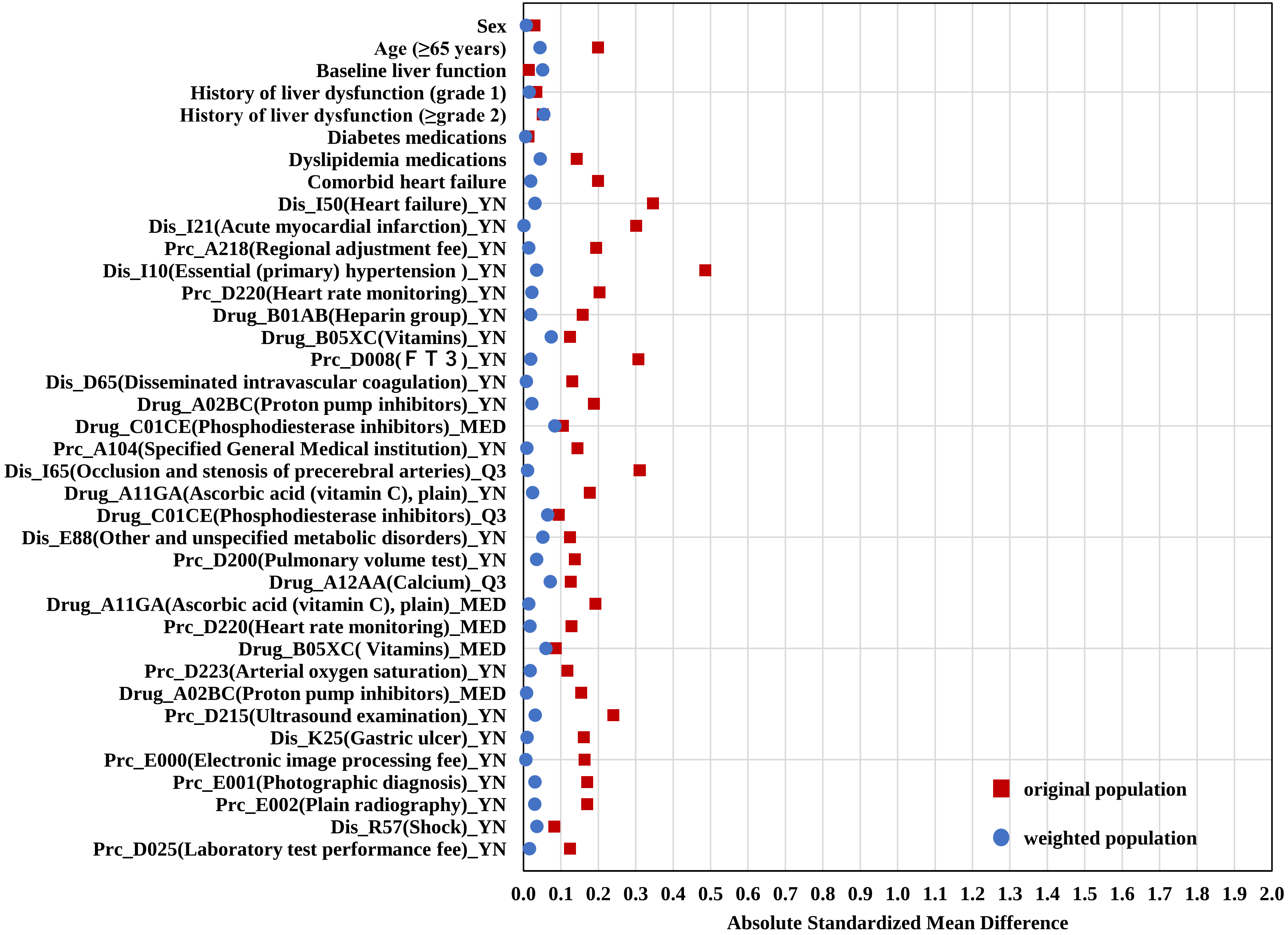

Supplemental Figure S3.5 Absolute standardized mean difference for basic covariates and top 30 covariates based on high-dimensional propensity score method comparing Exposure group 7 (Perindopril erbumine) to Control group (Enalapril maleate) in the original and the weighted population for the secondary outcome.
Dis, Disease; Prc, Procedure; YN, variable type (Yes or No); MED, variable type (<Median, Median≤); Q3, variable type (<Q3, Q3≤)
*The top 30 covariates are represented by “the dimension_code(description)_variable type”.

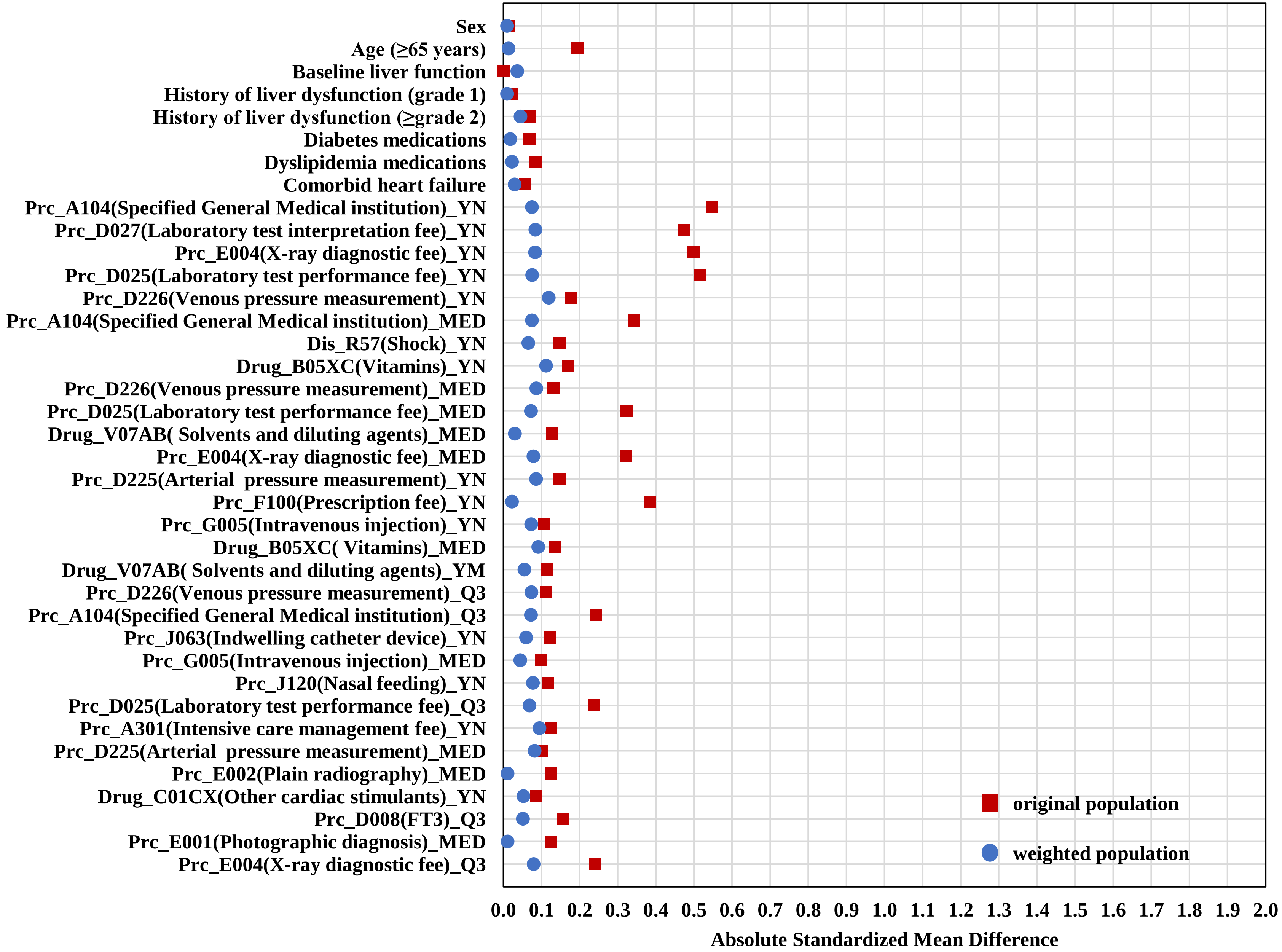

Supplemental Figure S3.6 Absolute standardized mean difference for basic covariates and top 30 covariates based on high-dimensional propensity score method comparing Exposure group 8 (Lisinopril hydrate) to Control group (Enalapril maleate) in the original and the weighted population for the secondary outcome.

Dis, Disease; Prc, Procedure; YN, variable type (Yes or No); MED, variable type (<Median, Median≤); Q3, variable type (<Q3, Q3≤)

*The top 30 covariates are represented by "the dimension_code(description)_variable type".

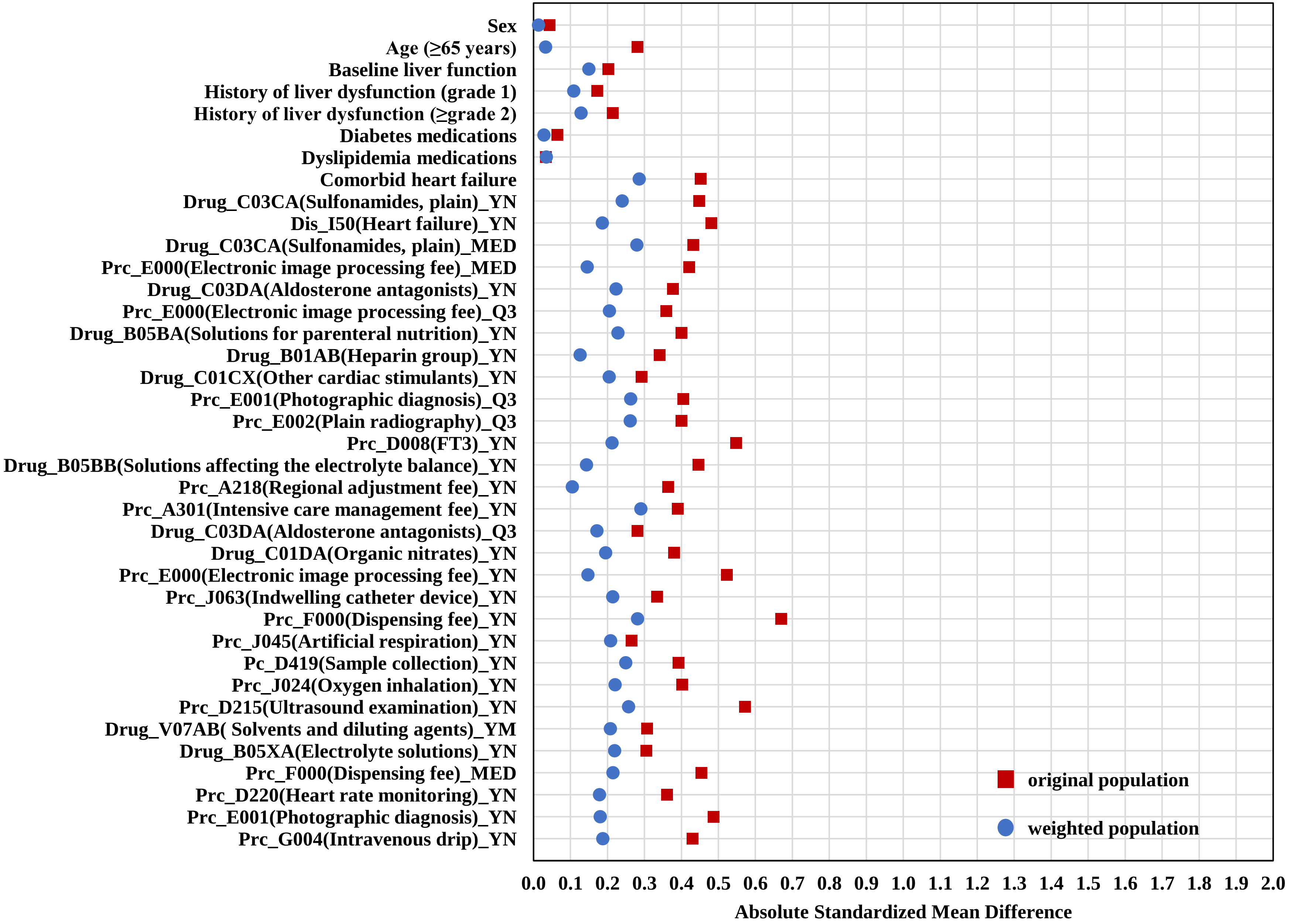

Supplemental Figure S3.7 Absolute standardized mean difference for basic covariates and top 30 covariates based on high-dimensional propensity score method comparing Exposure group 10 (Temocapril hydrochlori) to Control group (Enalapril maleate) in the original and the weighted population for the secondary outcome.
Dis, Disease; Prc, Procedure; YN, variable type (Yes or No); MED, variable type (<Median, Median≤); Q3, variable type (<Q3, Q3≤)
*The top 30 covariates are represented by “the dimension_code(description)_variable type”.

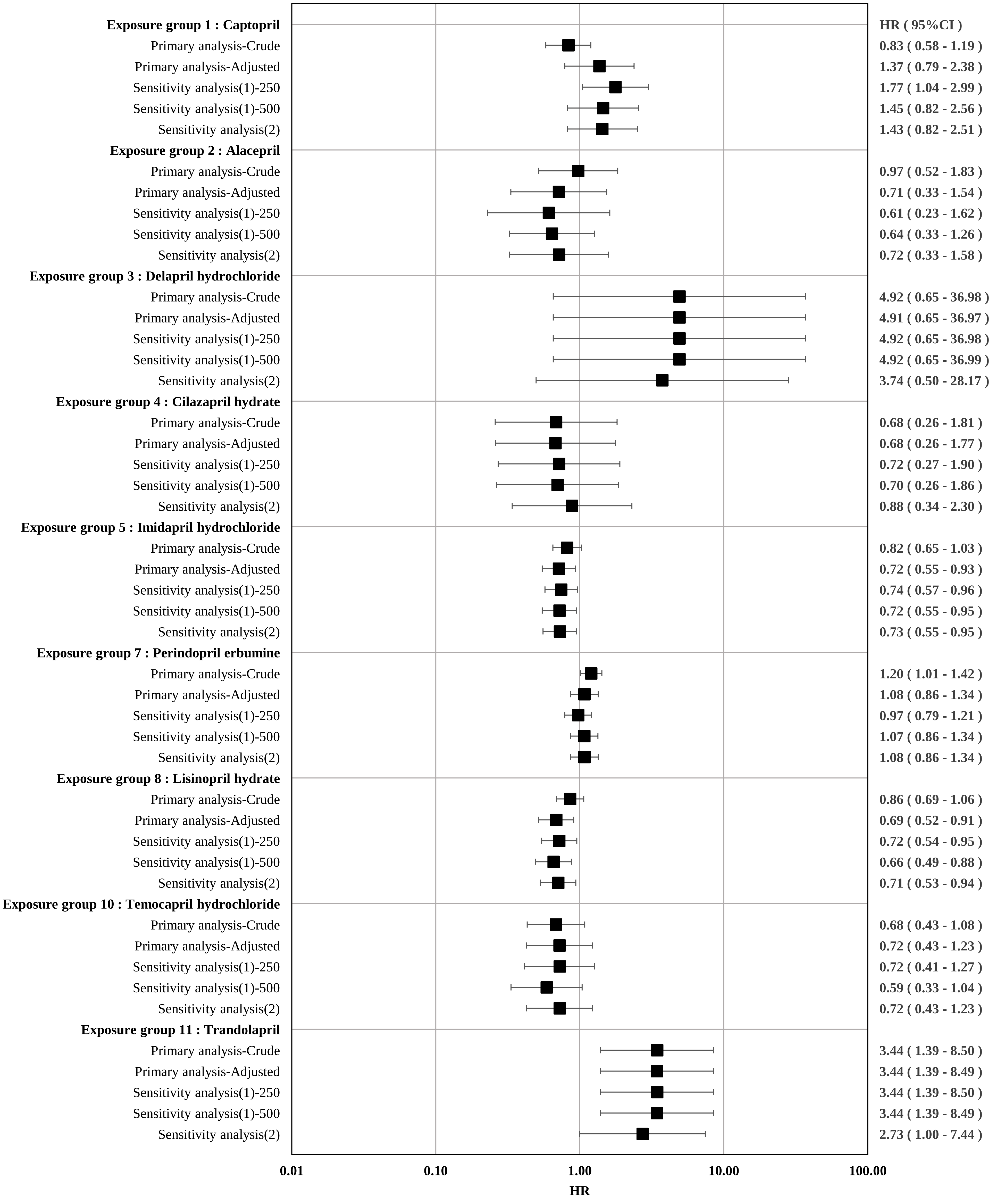

Supplemental Figure S4. Hazard ratios and 95% confidence intervals for each exposure group compared with control group (enalapril maleate) for primary outcome in the sensitivity analysis.

HR, hazard ratio; CI, confidence interval

Adjusted hazard ratios were estimated using inverse probability weighting based on the high-dimensional propensity scores calculated by logistic regression for each combination between the control and exposure groups. The number of selected covariates was as follows : Primary analysis-Adjusted and Sensitivity analysis(2) (one-tenth of the number of patients in the exposure group), Sensitivity analysis(1)-250 (250), Sensitivity analysis(1)-500 (500). Sensitivity analysis(2) was conducted restricted to populations with an overlapping the high-dimensional propensity score between the exposure and control group.

The estimation accuracy of the adjusted hazard ratio is low for groups with a small number of patients.

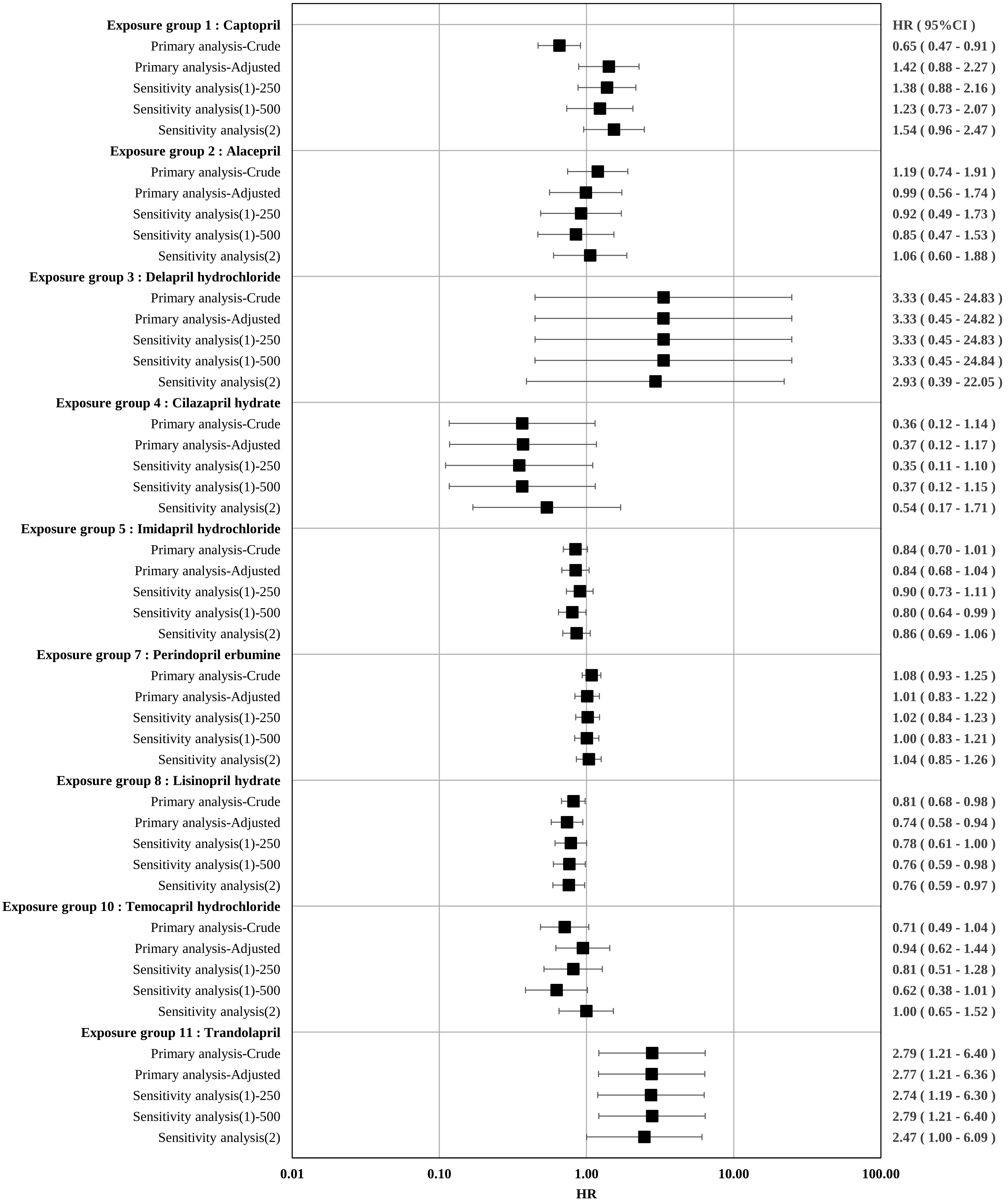

Supplemental Figure S5. Hazard ratios and 95% confidence intervals for each exposure group compared with control group (enalapril maleate) for secondary outcome in the sensitivity analysis.

HR, hazard ratio; CI, confidence interval

Adjusted hazard ratios were estimated using inverse probability weighting based on the high-dimensional propensity scores calculated by logistic regression for each combination between the control and exposure groups. The number of selected covariates was as follows : Primary analysis-Adjusted and Sensitivity analysis(2) (one-tenth of the number of patients in the exposure group), Sensitivity analysis(1)-250 (250), Sensitivity analysis(1)-500 (500). Sensitivity analysis(2) was conducted restricted to populations with an overlapping the high-dimensional propensity score between the exposure and control group.

The estimation accuracy of the adjusted hazard ratio is low for groups with a small number of patients.
